# Tipping points in urban growth: How urbanization reshapes flood resilience and social vulnerability in China

**DOI:** 10.1093/pnasnexus/pgag240

**Published:** 2026-07-09

**Authors:** Xiguang Liu, Yue Wu, Rui Ren, Hussam N Mahmoud

**Affiliations:** Institute for Resilient and Engineered Urban Planning, Institute for Interdisciplinary Innovation Research, Xi’an University of Architecture and Technology, Xi’an, Shaanxi 710055, China; Institute for Resilient and Engineered Urban Planning, Institute for Interdisciplinary Innovation Research, Xi’an University of Architecture and Technology, Xi’an, Shaanxi 710055, China; Institute for Resilient and Engineered Urban Planning, Institute for Interdisciplinary Innovation Research, Xi’an University of Architecture and Technology, Xi’an, Shaanxi 710055, China; Vanderbilt Center for Sustainability, Energy and Climate, Department of Civil and Environmental Engineering, Vanderbilt University, Nashville, TN 37235, USA

**Keywords:** urbanization, flood resilience, social vulnerability, coupling coordination

## Abstract

Urbanization is often portrayed as either a pathway to progress or a process that could amplify disaster risk. Yet, its true influence lies in how it reshapes resilience to hazards and the vulnerability of people most at risk. Using provincial data from China (2006–2020), we integrate urbanization, flood resilience, and social vulnerability into a unified framework to examine their coupled evolution. We find that rapid urbanization is a double-edged process: while it initially increases exposure, grouped cross-provincial analysis reveals a transition suggestive of a tipping point at the aggregate level, with social vulnerability declining sharply and resilience accelerating once the urbanization level enters a higher development stage (urbanization level > 0.75). Regional trajectories diverge, with provinces like Gansu trapped in vulnerability, Guangdong achieving resilience leaps, and Shanghai consolidating a high-level balance. Nationally, the coordination among the three systems rose by 83%, yet disparities persisted, particularly in western and northeastern regions. These findings show that the design of urban growth—not its pace alone—determines whether cities amplify or mitigate climate-driven flood risks, with lessons extending far beyond China.

Significance statement Rapid urbanization is often seen as the culprit behind increase in disaster risk, but its systemic impact on resilience and vulnerability still lacks a clear understanding. Our study reveals the nonlinear critical point at which urban growth shifts from amplifying risks to reducing risks; beyond this critical point, social vulnerability declines significantly, and flood resilience improves. We show that the quality and coordination of urban development shape flood risk trajectories more than exposure alone, and that different combinations of urbanization, resilience, and vulnerability generate distinct policy pathways. These insights provide practical guidance on how to steer urbanization to enhance climate resilience and reduce vulnerability.

## Introduction

More than half of humanity now lives in large cities, and urban growth is accelerating fastest in regions most exposed to climate extremes. Floods already affect more people worldwide than any other natural hazard, displacing millions of socially vulnerable populations annually and disrupting fragile economies (1). By mid-century, as sea levels rise and extreme rainfall intensifies, the number of urban residents exposed to flood risk could double. Yet, the outcome of urban growth is not predetermined: cities can either amplify disaster losses or enhance resilience, depending on how development is managed. The interplay between urbanization, resilience, and social vulnerability provides a conceptual basis for understanding this dual role of urban growth. Resilience refers to the capacity of urban systems to prepare for, absorb, recover from, and adapt to disruptive events such as floods (2), while social vulnerability refers to the socioeconomic and demographic conditions that shape the ability of populations to prepare for, respond to, and recover from disasters, such as age structure, employment, and so on (3).

Despite this urgency, most research treats the dynamics of urbanization, resilience, and social vulnerability in isolation or in pairs (4–6). Urbanization is often analyzed as a driver of exposure, resilience as a measure of infrastructure or governance robustness, and vulnerability as a social condition of inequality. But in reality, these three systems are deeply intertwined: the pace and design of urban growth shape resilience capacity; resilience investments can fail without attention to vulnerable groups; and vulnerability itself feeds back into urban systems by limiting adaptive capacity. Understanding how these systems evolve together—and whether tipping points exist that can shift cities from fragile to resilient—remains a critical gap in both science and policy.

Statistical evidence indicates that China is severely affected by floods globally, facing a persistently high risk of flooding (1). In years of severe flooding, the frequency is particularly pronounced: the number of flood events in major rivers can exceed 20, as demonstrated in 2020, when 21 major floods were recorded with sustained pressure across multiple river basins and time periods (7). Meanwhile, extreme rainfall in China has shown a marked upward trend, with rainstorm-induced floods becoming increasingly severe in recent years (8–10). Between 2006 and 2020, the economic risk exposure related to tropical cyclones shifted significantly northward, reflecting a reshaping of both the spatial distribution and exposure pattern of flood risk (11). Although the Chinese government has achieved notable progress in reducing casualties from a combination of engineering and nonengineering measures—such as reinforcing dams, upgrading drainage systems, and implementing early warning systems—the systemic nature of disaster risks continues to intensify (12, 13). This escalation arises from the compounded effects of climate change and expanding socioeconomic exposure (14, 15).

Flood impacts are shaped by interactions among urbanization, resilience, and social vulnerability. Urbanization can increase flood risk by expanding impervious surfaces, yet also improve infrastructure if well managed (16, 17). Jakarta illustrates how unplanned growth heightens vulnerability (18, 19), while Rotterdam shows how planning enhances resilience (20, 21). However, resilience remains uneven, with marginalized groups facing limited access to protection and recovery (22, 23). Thus, flood outcomes depend on integrating resilience and equity into urbanization processes.

The interplay between urbanization, resilience, and social vulnerability raises a critical question about how their interactions shape flood risk. In recent years, disaster risk research has increasingly incorporated the study of dynamic interaction mechanisms across multiple systems. Liu et al. (24) used a coupled coordination degree model to uncover the nonlinear interaction mechanism between urbanization and flood disasters. Similarly, Zhang et al. (25) highlighted that social structures can exert disruptive influences on the functioning of resilience systems. However, much of the existing literature primarily focuses on pairwise relationships. While some studies have proposed ternary analytical frameworks, many still remain confined to examining subsystems within a single dimension—such as dividing urban resilience into economic, social, and ecological components—in order to assess their internal coordination and adaptive capacity (26).

This study draws on statistical data from 31 Chinese provinces from 2006 to 2020 to construct a unified framework capturing how cities grow, withstand floods, and protect their most vulnerable residents. The framework is designed to assess development levels, trace spatiotemporal evolution, analyze interactions, and quantify the dynamic characteristics, spatial clustering patterns, and driving forces underlying their coupled coordination. The results offer a scientific foundation for advancing systemic flood risk management and strengthening resilience enhancement efforts. We develop an integrated framework examining urbanization, flood resilience, and social vulnerability across 31 Chinese provinces (2006–2020). Using a genetic-accelerated projection pursuit model and an improved coupling coordination degree (CCD) model, we identify nonlinear thresholds and regional pathways. Results reveal urbanization tipping points, persistent mismatches between resilience and vulnerability, and three development paths, offering insights for disaster risk science and urban climate adaptation.

## Results

**Figure 1:**
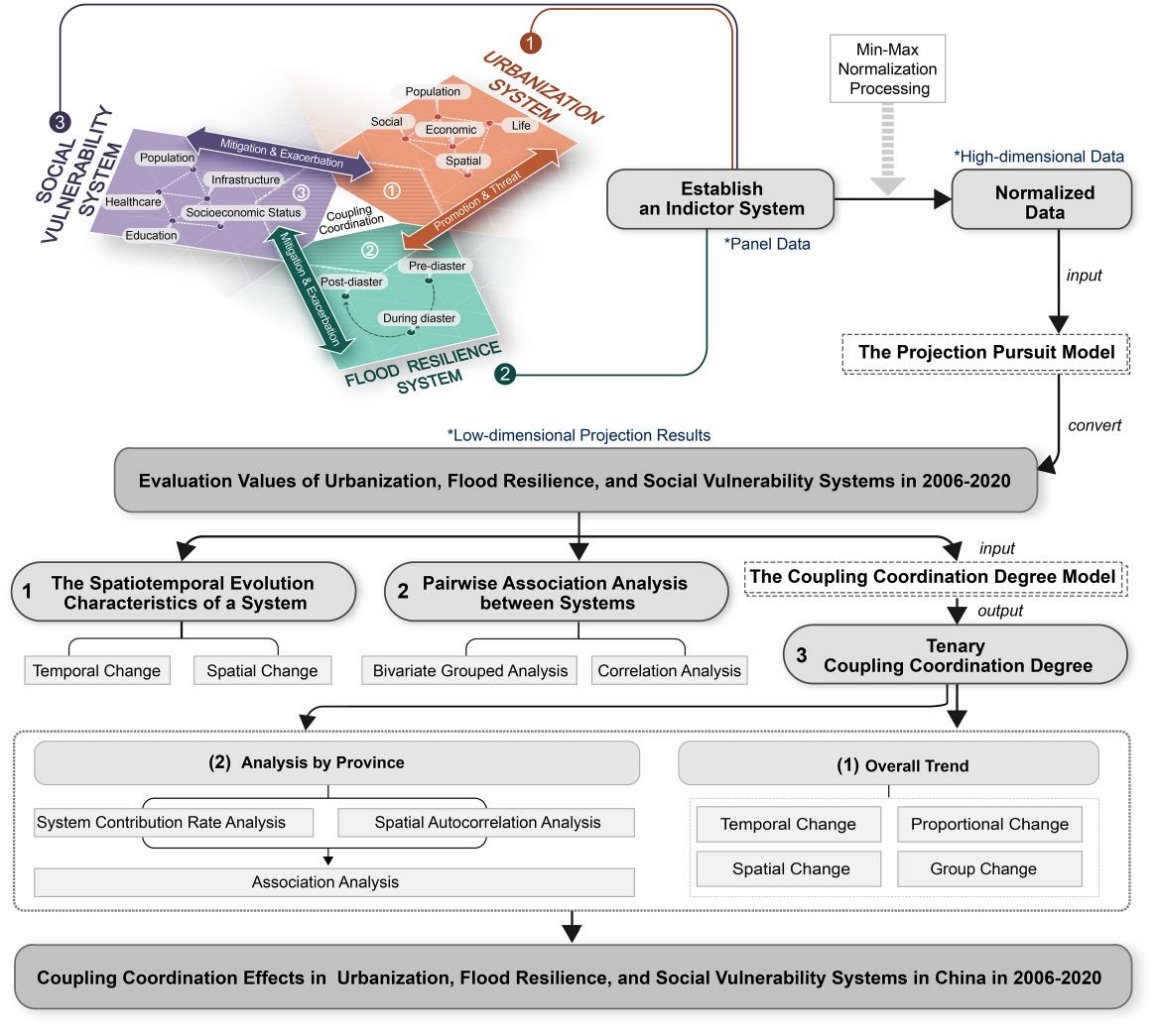
Theoretical framework of interaction among urbanization, flood resilience, and social vulnerability systems.

We contend that urbanization, flood resilience, and social vulnerability should be treated as the core analytical dimensions of this study because together they capture how flood risk is shaped by development patterns, adaptive capacity, and unequal social susceptibility. Other critical drivers of flood risk—including climatic trends, hazard intensity, and population distribution—are incorporated through the indicator framework of these three systems (summarized in Table 1 and listed in detail in Tables S1–S3), rather than treated as separate dimensions. A systemic understanding of flood risk therefore necessitates simultaneous attention to the interdependent dynamics of these three domains. Together, these factors constitute a feedback loop linking urbanization, resilience, and social vulnerability: urbanization influences exposure and shapes social structures, resilience defines the capacity for resistance and recovery, and vulnerability governs the amplification of losses. It is the dynamic coupling of the three systems that ultimately drives the spatiotemporal evolution of flood risk. The entire analysis approach is laid out in Fig. 1.

**Table 1:** Summary of urbanization, flood resilience, and social vulnerability system indices.

System	Content	Details
Urbanization System	Demographic urbanization	See Table S1
	Economic development	
	Spatial urbanization	
	Urbanization of society	
	Urbanization of life	
Flood Resilience System	Before the disaster (disaster prevention)	See Table S2
	During disasters (disaster intensity/communication stability)	
	Postdisaster (reconstruction and recovery)	
Social Vulnerability System	Consumption level	See Table S3
	Unemployment	
	Ethnicity	
	Age structure	
	Urban population ratio	
	Social security	
	Population growth	
	Education	
	Healthcare	
	Infrastructure	
	Transportation	

We first compiled statistical data on urbanization, flood resilience, and social vulnerability from 2006 to 2020, derived from national statistical yearbooks and public datasets (27–35). We observe that between 2006 and 2020, floods in China caused direct economic losses of nearly 3 trillion yuan, with 4.07 million houses damaged, 140 million hectares of farmland affected, and ∼1.6 billion people suffering losses in livelihoods and daily life (see more information in Fig. S1). In developing the indicator systems for urbanization, flood resilience, and social vulnerability, we draw on established studies while also considering data availability. Specifically, urbanization is measured across five dimensions—population, life, economy, society, and space—capturing both quantitative expansion and qualitative improvement of urban development (24, 36, 37). Flood resilience is based on the disaster risk management cycle, covering three stages: pre disaster, during disaster, and postdisaster response (24). The assessment of social vulnerability includes using widely recognized frameworks and focuses on five dimensions: population structure, healthcare, education, infrastructure, and socioeconomic status (3, 38).

**Figure 2:**
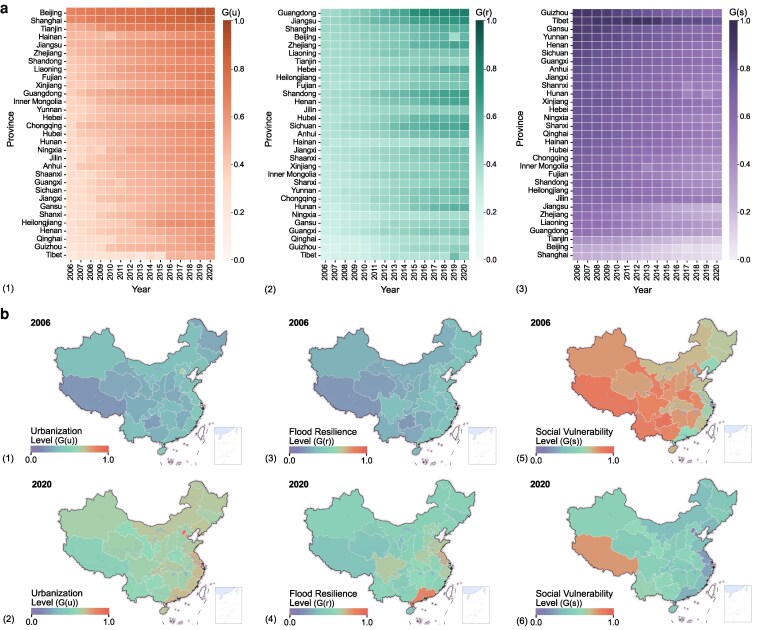
Changes in urbanization, flood resilience, and social vulnerability in China: a) provincial levels of the three systems. (1) Levels of urbanization; (2) Levels of flood resilience; (3) Levels of social vulnerability (see more information in Tables S1–S3). b) Spatial distribution changes in the three systems across China. (1) and (2) urbanization; (3) and (4) flood resilience; (5) and (6) social vulnerability (see Figs. S2–S4).

Based on this indicator framework, we use statistical data from 31 provinces in China from 2006 to 2020 (see more information in Data availability) and employ a projection pursuit model, optimized based on a genetic acceleration algorithm, to evaluate the development level of the three domains (see Methods). Figure 2a illustrates the temporal trends of urbanization (G (u)), flood resilience (G (r)), and social vulnerability (G (s)) in 31 provinces of China from 2006 to 2020. The results reveal marked differences in the development of these three dimensions, both over time and across provinces. Beyond temporal dynamics, we also examined their spatial distribution. As shown in Fig. 2b, the spatial patterns of urbanization, flood resilience, and social vulnerability in 2006 and 2020, highlight pronounced regional differences at different time points.

### Temporal changes in urbanization

Between 2006 and 2020, China's urbanization process showed both continuous overall progress and periods of slowdown, along with significant regional differences and a catch-up effect (Fig. 2a (1) and Table S4).

Viewed through China's 5-year planning cycles, urbanization shows three phases: rapid expansion (2006–2010, 6.88% annual growth), diffusion (2010–2015, 3.86%), and consolidation (2015–2020, 3.02%). Growth timing varied regionally, with most provinces peaking early, while others like Beijing and Shanghai peaked later. Meanwhile, urbanization advanced nationwide: provinces with an index above 0.5 increased from two in 2006 to all 31 by 2020, marking a broadly advanced stage of urban development.

From the perspective of regional balance, China's urbanization gap has narrowed during this period. The national average urbanization level rose from 0.358 to 0.656, with a growth rate of up to 83%. Meanwhile, the coefficient of variation—a measure of regional disparity—declined sharply from 0.288 to 0.120, and the range between the highest and lowest provinces contracted from 0.46 to 0.38. This catch-up effect is further supported by a strong negative correlation between urbanization growth rate and initial urbanization level (correlation coefficient = −0.72). Provinces with lower levels of urbanization in 2006, such as Heilongjiang, Guizhou, and Qinghai, experienced faster development, contributing to a more balanced spatial urbanization across China.

Despite the overall upward trajectory, challenges of unbalanced regional development remain. Although the ranking of most provinces changed little (Spearman rank correlation = 0.82), some experienced notable shifts. For example, Heilongjiang rose 15 places, demonstrating strong catch-up momentum, whereas Yunnan fell 15 places, reflecting a relative lag in its urbanization process. Overall, between 2006 and 2020, China's urbanization followed an evolutionary path of overall rise, regional catch-up, and phased slowdown. The national average kept increasing and regional disparities narrowed considerably; however, uneven growth across provinces underscore that urbanization still faces challenges of imbalance and differentiation.

### Temporal changes in flood resilience

Between 2006 and 2020, the level of flood resilience in Chinese provinces continued to improve, while the interprovincial gap gradually narrowed. Overall, flood resilience was characterized by a rising national average, uneven growth across provinces, and persistent spatial clustering (Fig. 2a (2) and Table S5).

From the perspective of macro development stages, combined with China's Five-Year plan framework, flood resilience experienced distinct phase-specific growth patterns. From 2006 to 2010, flood resilience experienced an initial growth stage, with an average annual growth rate of 3.85%. Between 2010 and 2015, growth accelerated, with the rate rising to 4.57%. From 2015 to 2020, flood resilience entered a consolidation stage, during which growth slowed to 2.11%. The difference in growth pace is also reflected across provinces: 18 provinces recorded their fastest resilience increase during the second stage, six provinces in the first stage, and only three provinces reached their growth peak in the third stage. During this period, the number of provinces exceeding a resilience threshold of 0.5 increased significantly. In 2006, no province reached this level. However, by 2020, 21 provinces had broken through this line, and in 2017, more than half of the provinces had achieved this level for the first time. These changes indicate that flood resilience in China had entered a more advanced stage of development.

From the perspective of regional balance, significant progress was made in flood resilience in various provinces of China, with the national average increasing from 0.335 to 0.540, representing a 61% increase. However, behind this overall improvement lies a complex pattern of rising averages with widening disparities. The range expanded from 0.21 to 0.43, and the standard deviation also increased from 0.059 to 0.097, indicating that improvements were more pronounced in some provinces than in others. In contrast, relative disparities remained stable: the coefficient of variation increased only marginally from 0.176 to 0.180, suggesting that flood resilience improved at broadly similar rates across provinces. Nevertheless, a small group of leading provinces advanced more rapidly, resulting in wider disparities despite generally synchronous nationwide progress.

Differences in growth pace among provinces further reshaped the relative distribution of flood resilience across China. The Spearman rank correlation coefficient between 2006 and 2020 is 0.64, indicating a moderate level of stability in the ranking, while also reflecting notable changes over time. For example, provinces such as Heilongjiang, Hunan, and Shandong rose substantially in the ranking, whereas Liaoning, Jilin, and Ningxia **decline in the ranking**. In summary, from 2006 to 2020, flood resilience of various provinces in China achieved an evolutionary trajectory of overall improvement, phased advancement, and widening absolute disparities. Although the national average increased steadily, the absolute gap between provinces widened, while relative disparities remained broadly stable. These findings suggest that, despite substantial nationwide progress, disparities between leading and lagging provinces persist and warrant continued attention.

### Temporal changes in social vulnerability

Between 2006 and 2020, the level of social vulnerability in Chinese provinces continuously declined, while the interprovincial gap showed a pattern in which national average decreased and disparities widened (Fig. 2a (3) and Table S6).

From the perspective of macro development stages, combined with China's Five-Year planning framework, social vulnerability improvement in various provinces showed phased changes. From 2006 to 2010, social vulnerability was in a declining phase with an average annual decrease of −2.60%. Between 2010 and 2015, social vulnerability entered an accelerated decline stage, with the rate reaching −3.96%. The period 2015 to 2020 was marked by another deaccelerated stage, where the annual rate of decline increased to 4.26%. During this period, the number of provinces with social vulnerability levels below 0.5 increased rapidly, from 3 in 2006 to 23 in 2020.

From the perspective of regional balance, social vulnerability significantly decreased, with the national average dropping from 0.708 to 0.419, a decrease of 40.9%, reflecting China's positive progress in reducing social vulnerability. At the same time, interprovincial disparities intensified: the range expanded from 0.656 to 0.706, and the coefficient of variation rose from 0.225 to 0.368. Compared with the overall decline across provinces, the pace of improvement became increasingly polarized, with provinces exhibiting higher initial levels of social vulnerability experiencing slower declines, while those with lower initial levels recorded more rapid reductions.

The differences in the decline pace among provinces further affect their relative position in the development of social vulnerability nationwide. The Spearman rank correlation coefficient between 2006 and 2020 is 0.9, indicating that the relative position between provinces remains basically unchanged, and the overall pattern did not experience fundamental reconstruction. In summary, from 2006 to 2020, social vulnerability in China followed a trajectory of overall decline, accelerated phases, and widening disparities. The national average significantly decreased, indicating an overall improvement. At the same time, both absolute and relative disparities expanded, while provincial rankings remained highly stable. This indicates that existing patterns were not fundamentally altered, and that highly vulnerable regions still need targeted, place-based measures to reduce vulnerability and prevent further divergence.

### Spatial distribution in urbanization

From 2006 to 2020, the spatial pattern of urbanization in China shifted from coastal polarization to regional balance. The mean values of the four major regions rose comprehensively, and the gap between the east and west converged from 0.17 to 0.12. Provinces generally crossed the 0.5 threshold, urbanization became more geographically widespread. As shown in Fig. 2b (1), in 2006, the spatial distribution was high in the east and low in the west. Beijing and Shanghai had the highest levels of urbanization, at 0.66 and 0.64, respectively, while Tibet had the lowest at only 0.20. The average urbanization level of the four major regions is as follows: 0.47 in the east, 0.30 in both the central and western regions, and 0.32 in the northeast.

As shown in Fig. 2b (2), by 2020 the high-value areas expanded into a “Bohai Rim–Yangtze River Delta–Pearl River Delta” corridor. From the perspective of urban agglomerations, urbanization first develops in core cities, and gradually spreads to surrounding areas in an orderly manner, ultimately forming cross-city collaborative development. The average urbanization levels in the Beijing–Tianjin–Hebei, Yangtze River Delta, and Pearl River Delta regions rose from 0.503, 0.513, and 0.380 to 0.760, 0.770, and 0.710, respectively. Inland urban agglomerations, including Chengdu–Chongqing, Guanzhong, and Jin–Shan drove the overall catch-up of central and western China. Overall, regional disparities in urbanization narrowed. Nevertheless, provinces such as Tibet, Gansu, Yunnan continue to exhibit low levels of urbanization, highlighting persistent regional differences.

### Spatial distribution in flood resilience

From 2006 to 2020, China's flood resilience improved, but also experienced structural differentiation. The overall enhancement is mainly reflected in the general improvement of the national resilience level. From the perspective of regional averages, the eastern, central, western, and northeastern regions all had higher averages in 2020 than in 2006. However, these gains did not translate into balanced regional resilience. Although the average gap between the east and west remained at 0.109, the growth rates varied significantly: the central region had the fastest improvement, followed by the western and eastern regions, while the northeastern region had the slowest improvement.

This unbalanced improvement in resilience led to structural differentiation in resilience patterns. As shown in Fig. 2b (3), in 2006, the pattern of strong east and weak west was relatively evident, and the high-resilience regions were mainly concentrated in the coastal core areas. However, as shown in Fig. 2b (4), by 2020 the pattern evolved into a new mode of coastal corridor, central anchors, and western overall uplift. Guangdong led with a resilience level of 0.83, while Jiangsu, Zhejiang, and Shandong jointly formed a continuous high-value belt. At the same time, the significant improvement in inland provinces such as Henan and Sichuan also formed new resilience anchor points. These findings indicate that, although flood resilience improved overall, leading provinces pulled further ahead, resulting in greater structural differentiation.

This phenomenon indicated that the overall improvement of China's flood resilience has not automatically resulted in complete regional balance. The spillover of the coastal corridor and the emergence of inland anchors reshaped the distribution of high-value areas, whereas relatively slow-growing regions, particularly provinces in Northeast China, still lag behind. In terms of policy, efforts should consolidate the diffusion effect of the corridor–anchor structure, while implementing differentiated strategies to compensate for the slowest-improving regions.

### Spatial distribution in social vulnerability

From 2006 to 2020, the spatial pattern of social vulnerability in China underwent a transformation from a simple polarization between coastal and inland regions to a complex differentiation of multiple levels and centers. As shown in Fig. 2b (5), in 2006, the social vulnerability in China was generally high, showing a clear gradient distribution of low along the coast and high inland. The eastern region had the lowest average at 0.563, while the western region had the highest average at 0.808. At that time, only Beijing, Shanghai, and Tianjin had vulnerability levels below 0.5.

As shown in Fig. 2b (6), this simple pattern discontinued in 2020. Coastal provinces entered a low-vulnerability stage, forming a stable cluster of low values. Meanwhile, several inland provinces, such as Sichuan and Chongqing, achieved significant improvements, which supported the overall decline in vulnerability in central and western China. The average values for central and western China decreased from 0.782 and 0.808 to 0.480 and 0.516, respectively. Highly vulnerable areas shrank from the original areas of Yunnan, Guizhou, Sichuan, Xizang, and Gansu to Tibet.

This evolution led to a new pattern: although the overall social vulnerability in China significantly declined, the mean gap between the eastern and western regions remained basically stable. There are differences in the rate of improvement among different regions, with the largest improvement being in the central region and the slowest in the northeast. This indicates that although the absolute level decreased, the relative gap between regions still needs to narrow further through more targeted strategies.

### Group comparisons among the three systems

**Figure 3:**
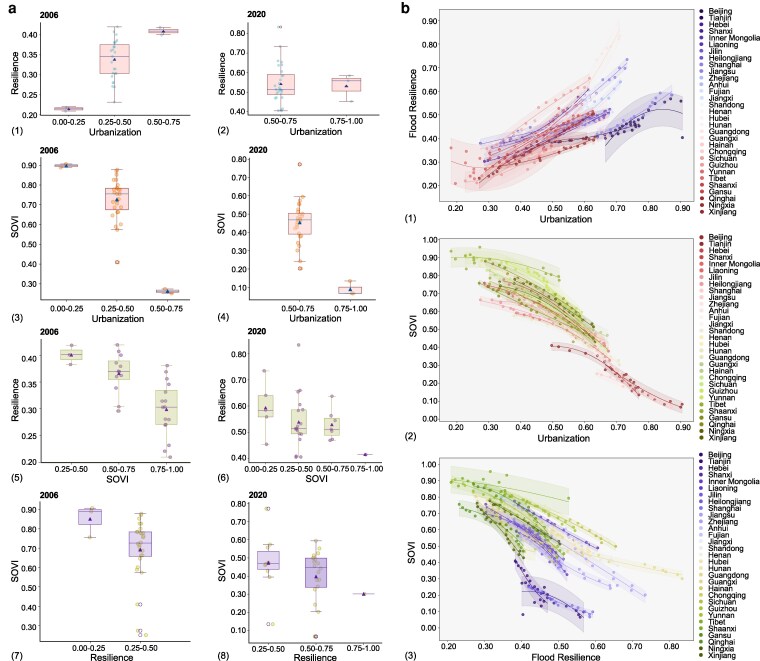
a) Group comparisons among the three systems for 2006 and 2020. (1) and (2) Resilience with urbanization; (3) and (4) Resilience with SOVI; (5) and (6) Resilience with SOVI; (7) and (8) SOVI with resilience. b) Relationships among the three systems in the 31 provinces. (1) Resilience with urbanization; (2) SOVI with urbanization; (3) SOVI with resilience (see more information in Figs. S5–S7).

The box plot shown in Fig. 3a is used to analyze the cross-sectional distribution of each province under specific grouping conditions. Unlike the previous analysis of spatiotemporal evolution, this method focuses on revealing intra group heterogeneity and the convergence or differentiation trends of provinces under similar levels of urbanization, flood resilience, or social vulnerability.

Figure 3a (1) and (2) illustrates the distribution of flood resilience in different provinces under different levels of urbanization in 2006 and 2020. Flood resilience is positively correlated with urbanization; however, the relationship exhibits a bottleneck at higher levels of urbanization, indicating that further urbanization yields smaller improvements in flood resilience. In 2006, the median flood resilience of different urbanization groups increased sequentially. By 2020, all provinces have surpassed the 0.50 threshold, with the median resilience reaching 0.51 in the 0.50–0.75 group and 0.56 in the 0.75–1.00 group. Meanwhile, interprovincial disparities (IQR) narrowed from 0.110 to 0.065. This suggests that as provinces reach a higher level of urbanization, the positive effect of further urbanization on flood resilience exhibits diminishing returns. Concurrently, the narrowing interprovincial disparities indicate a convergence in resilience levels.

Figure 3a (3) and (4) shows the distribution of social vulnerability in different provinces under different levels of urbanization in 2006 and 2020. Urbanization has a critical value for reducing social vulnerability, and there is a significant negative correlation between urbanization and social vulnerability. Data from 2006 showed that when the level of urbanization entered the 0.50–0.75 range, the median social vulnerability dropped significantly from 0.756 to 0.263. By 2020, this trend became more apparent. When urbanization remained within the 0.50–0.75 interval, the median vulnerability was 0.468. However, once urbanizationcrossed the critical threshold of 0.75, the median vulnerability plunged to 0.067, while interprovincial disparities (IQR) have also narrowed substantially to 0.034. This indicates that after reaching a certain level of urbanization, social vulnerability was rapidly reduced.

Figure 3a (5) and (6) shows the distribution of flood resilience in different provinces under different levels of social vulnerability in 2006 and 2020. Figure 3a (7) and (8) shows the distribution of social vulnerability in different provinces under different levels of flood resilience in 2006 and 2020. The grouping of social vulnerability shows a trend of differentiation from low to high levels of convergence. In 2006, the resilience level of various provinces was generally low and negatively correlated with social vulnerability. High vulnerability was accompanied by low resilience, and the median resilience showed a clear gradient across different vulnerability groups. But by 2020, with the overall improvement of resilience levels, the differences in resilience between different vulnerability groups significantly narrowed, with the median remaining in the range of 0.51–0.58, indicating that the improvement of flood resilience tends to be consistent among provinces.

Although improvement in flood resilience led to reduction in social vulnerability, the two were not completely synchronized. In 2006, provinces were mainly concentrated in the low resilience segment, corresponding to higher vulnerability. However, by 2020, the interprovincial difference in social vulnerability (IQR) in the high-resilience group was about 0.160, indicating a significant gap. These findings clearly illustrate a structural mismatch characterized by high resilience coexisting with persistent social vulnerability. This suggests that improvements in flood resilience alone are insufficient. Instead, resilience and social vulnerability should be addressed simultaneously through more integrated and coordinated policy interventions.

### Fitting relationships among the three systems

The fitting curve shown in Fig. 3b provides a quantitative perspective on the relationship between the three systems, revealing potential stable function relationships and nonlinear thresholds. Overall, urbanization is significantly positively correlated with flood resilience, with a Pearson correlation coefficient of 0.71. Urbanization is significantly negatively correlated with social vulnerability, with a Pearson correlation coefficient of −0.93. Flood resilience is moderately negatively correlated with social vulnerability, with a Pearson correlation coefficient of −0.64. These relationships indicate that the higher levels of urbanization are associated with greater flood resilience and lower social vulnerability. This pattern is consistent with the theoretical expectation that urbanization enhances flood resilience and reduces social vulnerability by improving public services and governance capabilities.

The quadratic fitting shows that there exists a significant nonlinear relationship among the three systems. By calculating the vertices of the fitted quadratic curve, we found that there are regional differences in these inflection points, which provides a more refined perspective for understanding the development path. As shown in Fig. 3b (1), there is an inverted U-shaped relationship between flood resilience and urbanization. The eastern and northeastern regions needed to reach a high level of urbanization (U ≈ 0.56–0.57) before resilience entered an accelerated improvement stage. In the central region, the phase of rapid improvement already started at a lower level of urbanization (U ≈ 0.39), showing a pattern of moderate urbanization level and rapid increase in resilience. In the western region, after reaching a moderate urbanization level (U ≈ 0.48), the growth rate of resilience slowed down, indicating the emergence of diminishing marginal returns. This heterogeneity demonstrates that the effectiveness of urbanization as a driver for flood resilience is highly contingent on the regional development stage.

As shown in Fig. 3b (2), social vulnerability decreases with increase in urbanization. However, the threshold at which vulnerability begins to decline varies across regions. In the eastern and northeastern regions, a relatively higher level of urbanization (U ≈ 0.52–0.53) is required to trigger this rapid decline in social vulnerability. In the central and western regions, the transition occurs at a lower urbanization level (U ≈ 0.41–0.44). As shown in Fig. 3b (3), social vulnerability decreased with increase in flood resilience. After flood resilience reached a threshold of ∼0.42–0.50, the effect of resilience on reducing vulnerability in the central, western, and northeastern regions continued to improve, whereas the eastern region has exhibited diminishing marginal returns. This indicates that in the high resilience stage, in addition to material construction, social policies need to be combined to further reduce vulnerability.

After confirming the significant correlations among the three systems, we adopted CCD as an indicator to assess the interaction and coordinated development level among two or more systems, elements, or variables (see Methods). CCD is commonly used to evaluate the synergistic effects between different systems and whether they can develop harmoniously. We calculated the ternary CCD values for 31 provinces in China from 2006 to 2020 (see more information in Table S7). Referring to previous studies (24, 26), we defined the range of 0.0–0.2 as seriously uncoordinated development, 0.2–0.4 as moderately uncoordinated development, 0.4–0.5 as slightly uncoordinated development, 0.5–0.6 as barely coordinated development, 0.6–0.8 as coordinated development, and 0.8–1.0 as superiorly coordinated development (see more information in Table S8).

### Temporal ternary CCD between the three system

**Figure 4:**
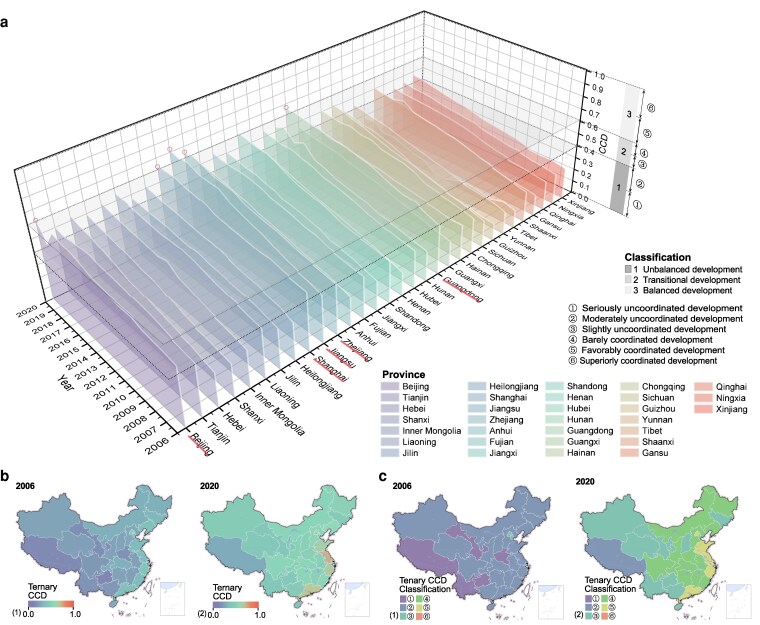
a) The CCD between urbanization, flood resilience, and SOVI in 31 provinces in China from 2006 to 2020. b) Spatial distributions of the values of ternary CCD in China in 2006 and 2020. c) Spatial distributions of the classification of ternary CCD in China in 2006 and 2020 (see more information in Figs. S8 and S9).

Figure 4a shows the temporal trend of ternary CCD in 31 provinces from 2006 to 2020. From 2006 to 2020, the ternary CCD in all provinces of China continued to increase significantly. The national average increased from 0.282 in 2006 to 0.517 in 2020, with a growth rate of 83.3%, shifting from moderately uncoordinated development to barely coordinated development. These results indicate a relatively low degree of coupling within the 0–1 range, which does not mean that urbanization, flood resilience, and social vulnerability are completely independent and unrelated. Instead, this means that there is still great room for coordinated development among the three in the future. The national average crossed 0.50 for the first time in 2019. Overall, although the average annual coefficient of variation of CCD values has decreased from 0.304 to 0.170, and the relative dispersion has converged, the range has expanded from 0.320 to 0.441. This indicates that while the overall coordination level improved, CCD increase for the top provinces is faster, thereby widening the gap between extreme values.

This increase was first led by the core coastal regions. The number of provinces with CCD values greater than 0.50 increased from 0 in 2006 to 20 in 2020. Shanghai first reached 0.50 in 2009, followed by Beijing in 2010, and Guangdong and Jiangsu in 2011. The provinces that reached 0.60 are also concentrated in these coastal areas: Jiangsu (2015), Guangdong (2016), Shanghai (2017), Zhejiang (2018), and Shandong (2019). A jump from 0.50 to 0.60 usually requires several years of accumulation, which reflects a staged threshold from barely coordinated development to coordinated development.

Driven by the regional frontrunners, inland provinces have also achieved significant catch-up. In terms of absolute growth, provinces such as Jiangsu, Henan, Sichuan, and Guizhou ranked among the top, with Guizhou showing a particularly outstanding performance with the highest growth rate of 217.8%. All provinces improved by at least one grade during this period, with five provinces advancing by three grades. This development pattern resulted in a certain continuity in the national CCD ranking (Spearman correlation coefficient = 0.742) but also reflects notable transitions.

### Spatial CCD between the three system

Figure 4b shows spatial trends in ternary CCD across 31 provinces (2006–2020). CCD improved nationwide, with rising averages in eastern, central, western, and northeastern regions; the central region saw the largest gain (0.275). Regional gaps narrowed slightly (east–west from 0.151 to 0.137) but persisted. In 2006, disparities were stark (Beijing 0.457 vs. Guizhou 0.137). By 2020, a clear pattern emerged in which coastal provinces led while inland provinces caught up, although western and northeastern provinces remained behind. Figure 4c shows spatial classification of ternary CCD in 2006 and 2020, revealing clear improvement. In 2006, most provinces were in low coordination stages. By 2020, developed regions (Yangtze River Delta, Pearl River Delta, and Beijing–Tianjin–Hebei) improved significantly, while western and northeastern provinces remained relatively low.

In summary, from 2006 to 2020, the overall ternary CCD of all provinces improved, showing a spatial evolution characterized by coastal regions leading and inland regions catching up. The regional gap narrowed but was not eliminated. At the same time, the overall level of inter provincial coordination improved in a step-wise manner, with coastal urban agglomerations taking the lead. However, some provinces in the western and northeastern regions were still relatively lagging behind.

### CCD classification and distribution trend analysis

**Figure 5:**
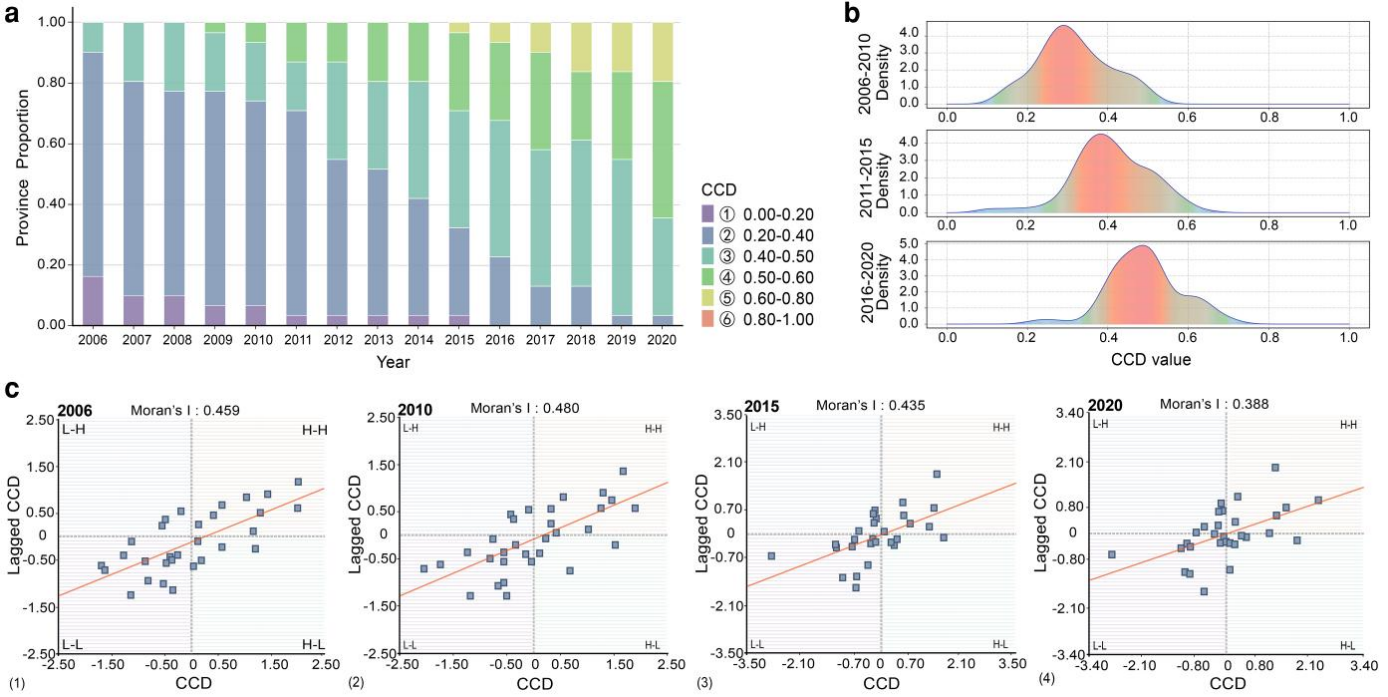
a) Proportional changes in provinces by CCD category. b) CCD grouping map. c) Clustering characteristics of 30 provinces (2006–2020); Hainan excluded due to queen adjacency. Lagged CCD denotes neighboring averages from a spatial weight matrix, used to reveal spatial dependence in Moran's I analysis (Figs. S10 and S11).

We calculated the changes in the proportion of provinces classified by different CCD each year. Figure 5a shows the trend of different CCD classification ratios in each province from 2006 to 2020. Between 2006 and 2020, there was a fundamental shift in the distribution of CCD levels across provinces in China.

In 2006, most provinces in China exhibited an uncoordinated development stage, with as many as 23 provinces in the moderately uncoordinated category, 5 provinces in the seriously uncoordinated category, and zero provinces having a CCD higher than 0.50. This distribution continued to migrate toward higher CCD levels over time and reached an important turning point in 2015. By 2015, the number of provinces with a CCD greater than 0.50 increased to nine, among which one province already entered the stage of superiorly coordinated development. Meanwhile, the number of provinces with a CCD less than 0.40 had significantly decreased to 10.

By 2020, provinces with high CCD levels became dominant, with 20 provinces having a CCD greater than 0.50, including 14 provinces in the barely coordinated development stage and 6 provinces in the coordinated development stage. At the same time, only one province remained in the moderately uncoordinated category, and the number of seriously uncoordinated provinces dropped to zero. This trend strongly indicates that the coordinated development level between urbanization, flood resilience, and social vulnerability in China continued to improve.

We further used Gaussian kernel density estimation (see Methods) to study the dynamic evolution of CCD values over a 15-year period, examining three 5-year plan periods from 2006 to 2010, 2011 to 2015, and 2016 to 2020. Figure 5b shows the kernel density distribution curves of CCD values for three sub-periods. We found that the dynamic evolution of CCD distribution exhibits a characteristic of overall rightward shift and eventual convergence.

During the period from 2006 to 2010, the distribution curve exhibited a unimodal pattern, with the peak concentrated in the 0.2–0.4 range, reflecting that most provinces were in the initial stage of moderately uncoordinated development. During the period from 2011 to 2015, the distribution curve showed a bimodal pattern. The main peak shifted to the right, indicating that the coordination level of most provinces had improved; however, the existence of the secondary peak also suggested that a few provinces remained in the uncoordinated development zone, presenting a clear polarization.

During the period from 2016 to 2020, the distribution curve evolved into a trimodal pattern. The main peak significantly shifted to the 0.5–0.6 range, with most provinces entering the transitional stage of barely coordinated development. Meanwhile, a small number of provinces in the 0.2–0.4 range and high-value provinces in the 0.6–0.8 range formed two secondary peaks, reflecting that some provinces achieved rapid growth in CCD levels in the later stage and entered a high-level coordinated development stage, while a few provinces experienced development divergence.

### Spatial clustering analysis of ternary CCD

As shown in Fig. 5c, the spatial clustering analysis of the ternary CCD of urbanization, flood resilience, and SOVI in China from 2006 to 2020 reveals significant regional differences and evolving spatial dependencies. In this analysis, provinces are categorized into four types—H–H (high–high), L–L (low–low), H–L (high–low), and L–H (low–high)—indicating spatial clustering of high and low coordination levels across different regions (see Methods). The global spatial autocorrelation (Moran's I) of the ternary CCD was positive between 2006 and 2020, indicating significant spatial clustering across the country. The number of core clusters (H–H and L–L) decreased from 22 provinces in 2006 to 17 in 2020, while transitional provinces (H–L and L–H) increased from 8 to 13, suggesting a blending of original cluster boundaries: high-value influences have spilled over to surrounding areas, and broader transition zones have emerged between regional blocks.

More specifically, in 2006, the high-value cluster (H–H) included 9 provinces, forming a coastal high-value core; the low-value cluster (L–L) included 13 provinces, mainly distributed across the southwest, northwest, and parts of the central-northern regions. There was almost no change in 2010. In 2015, the number of low-value with high-value neighbors (L–H) increased to 6 provinces, indicating an expansion of the transition zones between low-value provinces and their high-value neighbors. By 2020, the H–H cluster shrank to 6 provinces, and the L–L cluster had reduced to 11 provinces, with their spatial distribution mainly concentrated in the northwest and southwest. Meanwhile, the number of H–L and L–H transitional provinces increased. These changes reflect a shift from continuous high-value coastal clusters to a point–belt structure, with weakened homogenous clustering and enhanced boundary blending.

### Evolution analysis of provincial ternary CCD from 2006 to 2020

**Figure 6:**
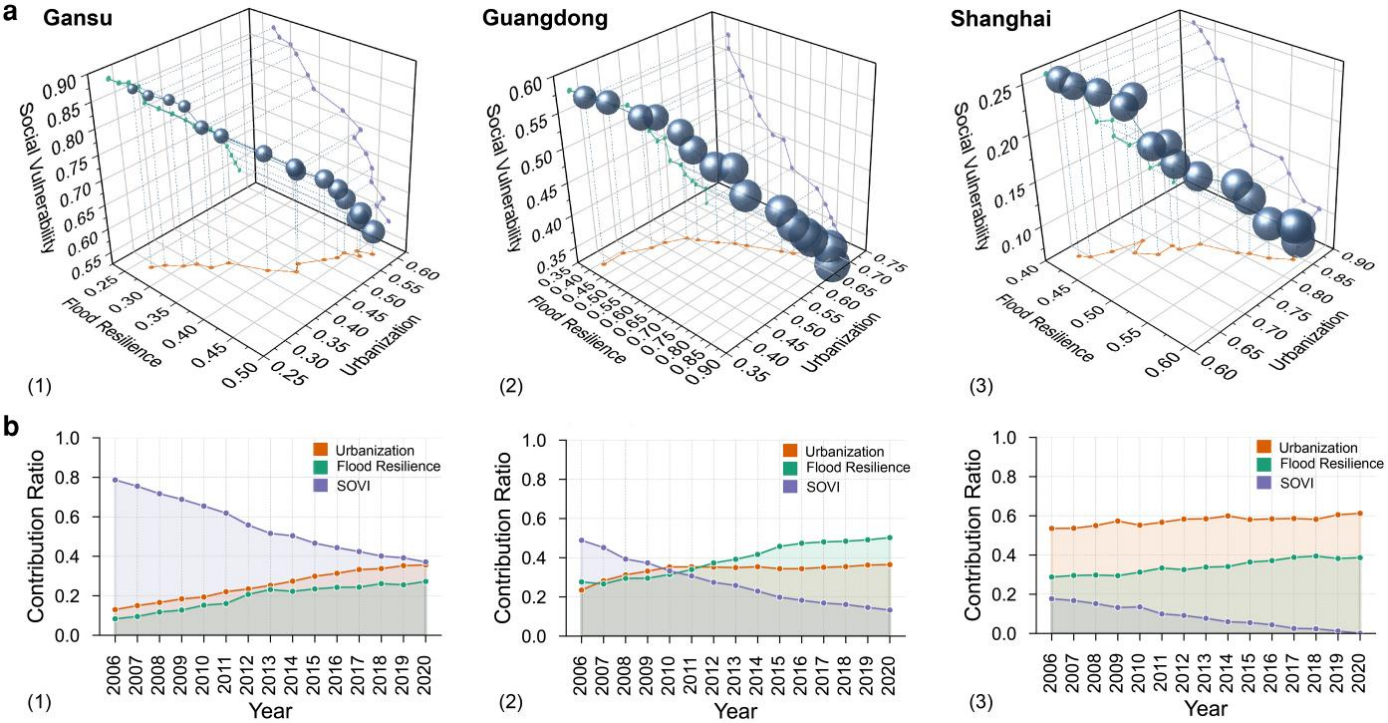
a) Provincial evolution of the ternary CCD among urbanization, flood resilience, and social vulnerability in Gansu, Guangdong, and Shanghai from 2006 to 2020 (see more information in Fig. S12). b) Changes in contribution rates of the three systems in Gansu, Guangdong, and Shanghai from 2006 to 2020 (see more information in Fig. S13).

To depict the coevolution of urbanization (U), flood resilience (R), and social vulnerability (S), we plotted the development trajectories of each province in 3D space (Figs. 6a and S12). Sphere size represents the ternary CCD, with larger spheres indicating higher coordination. Figure 6b shows annual contributions of each system. Gansu, Guangdong, and Shanghai illustrate three typical paths: S-dominated, R-dominated, and U-dominated, respectively.

Gansu represents an early-stage urbanization path with high vulnerability and persistently low CCD. In Fig. 6a (1), spheres remain small with CCD below 0.5 even in 2020. Contribution analysis (Fig. 6b (1)) shows social vulnerability to consistently dominate, though gradually declining. Spatial clustering (Fig. 5c) places Gansu mainly in the L–L cluster. Thus, coordinated development should prioritize reducing social vulnerability, followed by improving urbanization and resilience.

Guangdong's represents an accelerated take-off model driven by postdisaster governance. In Fig. 6a (2), its trajectory shifts rapidly from moderate U-R and high S to high U-R and low S, with rising CCD. Figure 6b (2) shows a dominance shift (2009–2012): social vulnerability led initially, then resilience became dominant. This demonstrates a disaster-learning reinforcement loop, where repeated events enhance governance and coordination.

Shanghai represents a mature path. In Fig. 6a (3), it begins with high U, high R, and low S, maintaining high and steadily increasing CCD. Figure 6b (3) shows urbanization as the main contributor, declining social vulnerability, and rising resilience. This indicates a shift from scale-driven growth to structural optimization, consistent with the plateau effect in Fig. 3.

Overall, while CCD improved across provinces, pathways differed. Gansu started with vulnerability reduction first; Guangdong depended on resilience breakthroughs after urbanization gains; Shanghai relied onfine-tuning flood resilience and maintaining low vulnerability at a high-development platform.

These three development paths offer a micro-level interpretation of the national evolutionary pattern revealed in Figs. 4 and 5, characterized by coastal core dominance, inland diffusion, and western catch-up. They also provide insights for formulating differentiated regional policies: the western region should prioritize reducing social vulnerability and providing basic guarantees; coastal areas should continue to upgrade their resilience; and mega city clusters should focus on efficiency improvement and refined governance.

### Dominant system evolution

**Figure 7:**
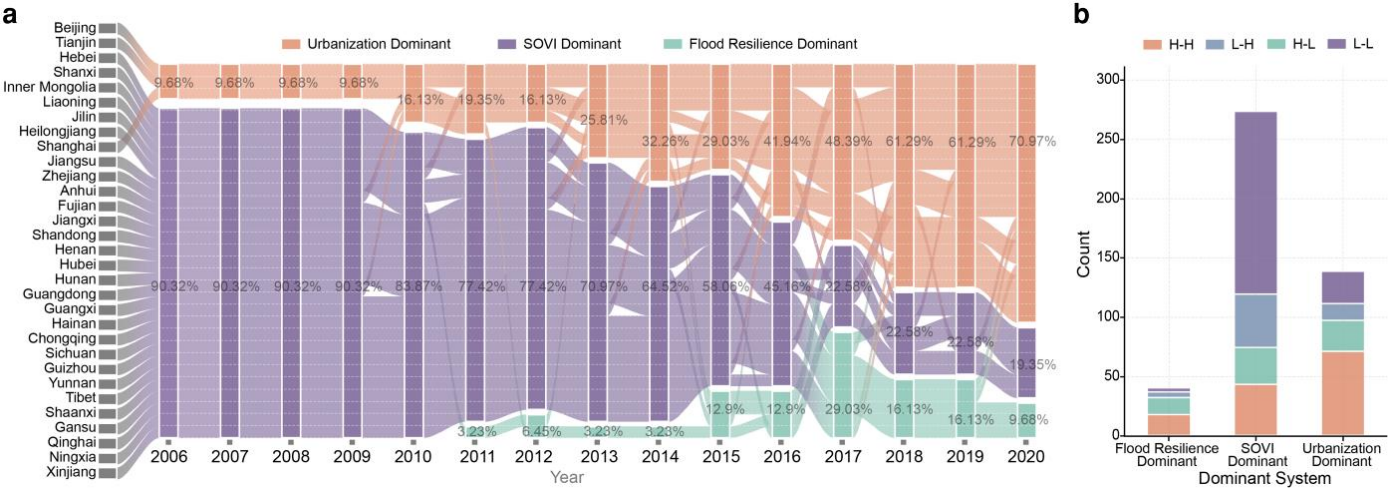
a) Evolution of dominant systems across provinces, 2006–2020 (see more information in Table S9). b) Distribution of High-High, High-Low, Low-High, and Low-Low clusters by dominant systems.

Figure 7a illustrates the temporal evolution of the dominant systems at the provincial level. Between 2006 and 2020, there was a significant and clear shift in the dominant system of CCD in various provinces of China. In 2006, 90.32% of provinces were dominated by social vulnerability, while only 9.68% were dominated by urbanization, and flood resilience had not yet become the dominant system.

Since then, the proportion of provinces dominated by social vulnerability declined year by year: 83.87% in 2010, 58.06% in 2015, and down to 19.35% by 2020. At the same time, the proportion of provinces dominated by urbanization continued to rise, starting from 9.68% in 2006, approaching social vulnerability-dominated provinces for the first time in 2015, and reaching a turning point in 2017, accounting for 48.39%, surpassing social vulnerability-dominated and flood resilience-dominated provinces for the first time. By 2020, urbanization-dominated provinces further increased to 70.97%, becoming the most prevalent dominant type nationwide. Flood resilience dominance first emerged in 2011, peaked at 29.03% in 2017, and then declined to 9.68% by 2020.

From the perspective of inter provincial changes, between 2006 and 2020, a total of 19 provinces shifted from being dominated by social vulnerability to being dominated by urbanization, and 6 provinces shifted from being dominated by social vulnerability to being dominated by flood resilience. Only three provinces—Yunnan, Tibet, and Gansu—remained consistently dominated by social vulnerability, whereas Beijing, Tianjin, and Shanghai were always dominated by urbanization. Overall, the dominant type presents a clear path of social vulnerability gradually receded, urbanization became increasingly dominant, and flood resilience steadily gained prominence.

Figure 7b presents a paired analysis between dominant systems and spatial clustering from 2006 to 2020, revealing a clear association between the two. Provinces dominated by urbanization appear more frequently in high-value or transitional clusters: they appear 71, 26, 14, and 27 times in the H–H, H–L, L–H, and L–L clusters, respectively. This indicates that regions with higher levels of coordination are typically dominated by urbanization. The dominance of social vulnerability is highly correlated with the L–L clustering, appearing 153 times. Although social vulnerability dominance also appears in other clusters, its high concentration in the L–L cluster—accounting for 56.3% of all its occurrences—indicates that socially vulnerable regions tend to be geographically concentrated and generally exhibit low CCD levels.

Flood resilience-dominated provinces are few and cluster mainly in high or transitional contexts: 18 in H–H, 14 in H–L, 5 in L–H, and 3 in L–L. Resilience dominance aligns with improving environments, urbanization with higher-level clusters, while vulnerability dominance remains concentrated in L–L, reflecting spatial restructuring patterns.

## Discussion

Between 2006 and 2020, China's provinces improved coordination among urbanization, flood resilience, and social vulnerability, reflected in rising CCD. However, disparities persist: coastal and central regions show high coordination, while western and northeastern areas lag, consistent with evidence linking uneven resilience capacity to economic development, infrastructure, and governance differences (39, 40).

A key insight from our analysis is the existence of tipping points in urban development. Once urbanization surpassed a critical threshold, vulnerability declines steeply and resilience accelerates, revealing a nonlinear pathway by which equitable and well-planned cities can escape cycles of risk. Conversely, resilience investments that are not paired with reductions in vulnerability create “structural mismatches” where improved infrastructure coexists with entrenched social fragility. Such structural mismatches have been documented elsewhere: in rapidly urbanizing cities, unequal allocation of adaptation resources has been shown to reduce flood risk in some areas while leaving socially vulnerable groups disproportionately exposed, confirming that infrastructure-oriented resilience without accompanying social equity achieves only partial adaptation (41, 42).

These findings point to a historic shift in governance priorities. In 2006, social vulnerability-dominated flood outcomes across most provinces; by 2020, urbanization had become the primary driver of coordination. This transition suggests that the quality of urban planning and governance now exerts greater influence on risk trajectories than demographic or hazard exposure alone. The implication is that cities are not simply victims of climate change—they are arenas where resilience can be actively designed.

The differentiated development paths of Gansu, Guangdong, and Shanghai highlight policy pathways relevant beyond China. Vulnerability-dominated regions require immediate expansion of social protection and healthcare to create a foundation for resilience. Resilience-dominated regions demonstrate the value of postdisaster learning loops, where repeated shocks accelerate governance capacity through iterative learning, cross-sector collaboration, and multilevel coordination (43). Urbanization-dominated megacities reveal the challenges of a high-level platform, where further progress depends not on expansion but on structural optimization, redundancy, and social equity.

From a global perspective, these results speak to pressing challenges in other rapidly urbanizing regions of Asia, Africa, and Latin America. As climate extremes intensify, unplanned growth risks deepening inequality and entrenching vulnerability. Yet when urbanization is paired with inclusive governance and risk-sensitive planning, it can trigger resilience tipping points that reduce vulnerability across entire regions.

## Conclusions

This study provides the first provincial-scale, longitudinal assessment of the coupling coordination among urbanization, flood resilience, and social vulnerability in China from 2006 to 2020. The results reveal a substantial improvement in the coordination among the three systems over the study period, indicating that China's urban development has increasingly moved toward a more integrated relationship with flood resilience and social vulnerability reduction. We also find that the relationship between urbanization and flood resilience is nonlinear, with a tipping-point-like transition emerging around an urbanization level of 0.75. In most provinces, beyond this point, flood resilience tends to improve more rapidly, while social vulnerability declines more substantially. Spatially, provinces follow heterogeneous pathways of urban growth, resilience enhancement, and vulnerability reduction, reflecting distinct regional combinations of development stage, governance capacity, and social susceptibility.

Three contributions distinguish this work from prior disaster risk research: (1) methodologically, we operationalize coupling coordination analysis for tri-system interactions rather than conventional dual-system couplings; (2) empirically, we document a governance-priority inversion in which urbanization has overtaken social vulnerability as the dominant driver of flood risk outcomes; and (3) theoretically, we introduce a three-pathway typology—vulnerability-dominated, resilience-dominated, and urbanization-dominated—offering a transferable lens for comparative urban climate research.

For policymakers, the lesson is clear: urbanization is not destiny, but design. Investments in gray-green infrastructure, equitable service provision, and social protection can transform cities from amplifiers of flood risk into buffers against climate extremes. Embedding these principles in international frameworks such as the Sendai Framework and the Sustainable Development Goals will be critical for translating local successes into global resilience.

Finally, while our study is limited by its provincial scale and reliance on proxy indicators for hazard exposure, future work integrating high-resolution climate data and microlevel social surveys could deepen understanding of these dynamics. A further limitation is that some indicators may have alternative conceptual classifications. For example, population density and per capita area of urban roads are included in our indicator framework as part of the urbanization system because they primarily represent demographic concentration and spatial urbanization. However, these indicators also influence flood exposure and could therefore be considered relevant to resilience assessment, and their classification may affect the interpretation of the observed relationship between urbanization and flood resilience. Future research could test the robustness of this relationship by reclassifying such exposure-related indicators or by introducing exposure as a separate analytical dimension. Extending analyses beyond floods to heatwaves, droughts, and compound hazards will further test whether the resilience–urbanization–vulnerability relationship observed in China represents a broader law of urban climate adaptation.

## Materials and methods

### Index system

The coupling among urbanization, flood resilience, and social vulnerability underpins coordinated urban development. CCD quantifies their interactions. We constructed an indicator system: urbanization includes five dimensions (economic, living, demographic, social, spatial; Table S1); flood resilience covers pre-, during-, and postdisaster stages (Table S2); and social vulnerability spans five types (Table S3). A database was compiled for 31 provinces from 2006 to 2020 with annual indicator data (Data availability).

### Assessment of urbanization, flood resilience, and SOVI systems

The projection pursuit model was used to determine the comprehensive levels of urbanization, flood resilience, and social vulnerability systems. This model is a statistical technique used for data analysis. It can project high-dimensional sets onto low-dimensional sets to uncover structures or features within the projected space (24). The computational process is divided into the following three steps:

1. Data preprocessing

This step involves the normalization of the sample evaluation index set. Assume the sample set of index values is $\left\{x^{*}(i,j)|i=1\cdots n,j=1\cdots p\right\}$, where $\;x^{*}(i,j)$ is the *j*th index value of the *i*th sample, with *n* and *p* representing the number of samples (sample size) and the number of indexes, respectively. To eliminate the dimensions of the index values and unify the range of variation of the index values, extreme value normalization is employed.

For indicators where higher values are better, the normalization process is as follows:


(1) $$x(i,j)=\frac{x^{*}(i,j)-x_{\min}(j)}{x_{\max}(j)-x_{\min}(j)}.$$

For indicators where lower values are better, the normalization process is as follows:


(2) $$x(i,j)=\frac{x_{\max}(j)-x^{*}(i,j)}{x_{\max}(j)-x_{\min}(j)},$$

where $x_{\max}(j)$ and $x_{\min}(j)$ are the maximum and minimum values of the *j*th indicator, respectively, and x(i,j) is the sequence of normalized indicator values.

2. Determining the projection function

The Projection Pursuit method synthesizes P-dimensional data $\{x^{*}(i,j)|i=1\cdots n,j=1\cdots p\}\;$ into a 1D projection value G(i) with a={a(1),a(2),a(3),⋯,a(p)} as the projection direction:


(3) $$G(i)=\overset{p}{\underset{\;j=1}{\sum }}\;\alpha jx(i,j),(i=1\cdots n).$$

The projection function is as follows:


(4) $$Q(a)=S_{g}D_{g},$$

where $S_{z}$ is the SD of the projection values G(i); $D_{z}$ is the local density of the projection values G(i).


(5) $$S_{g}=\sqrt{\frac{\sum_{i=1}^{n}\;{\left(G(i)-E(G)\right)}^{2}}{n-1}}$$

(6) $$D_{g}=\overset{n}{\underset{i=1}{\sum }}\;\overset{n}{\underset{\;j=1}{\sum }}\;(R-r(i,j))*u(R-r(i,j)),$$

where E(G) is the mean value of the sequence {G(i)|i=1⋯n}; *R* is the window radius for local density. The selection of *R* should ensure that the average number of projection points within the window is not too small to avoid excessive sliding bias, and it should not increase too much with *n*. *R* can be determined experimentally, and it is generally set to 0.1 $S_{z}$; r(i,j) represents the distance between samples, defined as r(i,j)=|G(i)−G(j)|; u(t) is a unit step function where u(t) equals 1 when t ≥ 0 and where *u(t)* equals 0 when t < 0.

3. Calculating the projection values

A genetic algorithm was used to improve the projection index function Q(a), and the projection value G(i) was calculated. The integrated level of the urbanization and flood resilience systems was shown using projection values (comprehensive levels).


(7) $$\max Q(a)=S_{g}\cdot D_{g}$$

(8) $$s.t.\overset{p}{\underset{\;j=1}{\sum }}\;a^{2}(j)=1$$

The comprehensive level evaluation of urbanization, flood resilience, and SOVI was obtained through the above calculations (see more information in Tables S4–S6).

### Improved CCD model

Our study adopted an optimized ternary CCD model. Its advantage lies in increasing the discriminative power of *C* values, dispersing *C* as evenly as possible in (0,1), and improving the validity of the model. Based on this optimized coupling coordination model, further calculations can more reasonably represent the coupling coordination relationship (24).


(9) $$C=\sqrt{\left[1-\frac{\sum_{i>j,j=1}^{n}\;\sqrt{{\left(U_{i}-U_{j}\right)}^{2}}}{\sum_{m=1}^{n-1}\;m}\right]\times {\left(\overset{n}{\underset{i=1}{\prod }}\frac{U_{i}}{\max U_{i}}\right)}^{\frac{1}{n-1}}}$$

(10) $$T=\overset{n}{\underset{i=1}{\sum }}\;\alpha_{i}\times U_{i},\overset{n}{\underset{i=1}{\sum }}\;\alpha_{i}=1$$

(11) $$D=\sqrt{C\times T},$$

where $U_{i}$ is the evaluation value of the *i* system; $\alpha_{i}$ is weight of the *i* system; $U_{i}\in [0,1]$, C∈[0,1], the more discrete the systems are, the lower the *C* value; on the contrary, the higher the *C* value.

As our study explores the ternary coupling and coordination relationship between urbanization, flood resilience, and social vulnerability system, therefore, when *n* = 3, assuming max $U_{i}$ is $U_{3}$


(12) $$\begin{array}{rl} C\;= & \sqrt{\left[1-\frac{\sqrt{{\left(U_{3}-U_{1}\right)}^{2}}+\sqrt{{\left(U_{2}-U_{1}\right)}^{2}}+\sqrt{{\left(U_{3}-U_{2}\right)}^{2}}}{3}\right]}\\ & \times \sqrt{\frac{U_{1}}{U_{3}}\times \frac{U_{2}}{U_{3}}} \end{array}$$

(13) $$T=\alpha_{1}U_{1}+\alpha_{2}U_{2}+\alpha_{3}U_{3},\;\alpha_{1}+\alpha_{2}+\alpha_{3}=1$$

(14) $$D=\sqrt{C\cdot T},$$

where $U_{1}$, $U_{2}$, and $U_{3}$ are the comprehensive levels of urbanization, flood resilience and social vulnerability systems ($U_{1},\;U_{2},\;\mathrm{and}\;U_{3}\;$ can be substituted with the previously obtained G(i) value); while *C* represents the coupling coefficient between them; *T* is the comprehensive level of system; *α* indicate the contribution coefficients of urbanization and flood resilience. It is considered that flood resilience system is as significant as urbanization subsystem in China, social vulnerability system is as significant as urbanization subsystem in China, i.e. $\alpha_{1}\;=\;\alpha_{2}\;=\;\alpha_{3}\;=\;\frac{1}{3}$; and *D* represents the CCD. The CCD may be split into three classes based on the degree of interaction between $U_{1}$, $U_{2}$, and $U_{3}$ (see more information in Table S8).

### Spatial autocorrelation

Using Local Moran's *I*, the spatial clustering effects of CCD across provinces was examined. Moran's *I* > 0 indicate positive clustering, < 0 negative correlation, and 0 randomness. ArcGIS linked CCD values to provincial units and calculated global Moran's *I* of 0.206, indicating positive spatial autocorrelation. The expected value (−0.030), variance (0.006967), Z-score (2.84), and *P*-value (0.0046) confirm statistical significance (Fig. S10).

Next, we used GeoDa software to analyze the local spatial autocorrelation of CCD values for 31 provinces in China, resulting in four types of clusters. We categorized the clustering patterns of the 31 provinces based on their relationships with adjacent provinces into four types: H–H, L–L, L–H, and H–L (see more information in Fig. S11).

### Gaussian kernel density estimation

Gaussian Kernel Density Estimation applies a Gaussian (normal) distribution “kernel” to each data point, and then sums all the kernel functions to form an overall density estimation curve, helping us understand the data distribution pattern. In Kernel density estimation, $D_{1}$, $D_{2}$, …, $D_{n}$ are *n* samples for the CCD of urbanization–flood resilience system. The probability density function of urbanization–flood disasters system was defined as f*(D).


(15) $$f\;*(D)=\frac{1}{nh}\overset{n}{\underset{i=1}{\sum }}\;K\left|\frac{D_{i}-D}{h}\right|,$$

where *n* is the number of samples, *h* is the bandwidth, and *D* is the average value. *K* is the kernel function, which usually describes a symmetric single-peak probability density function centered on 0.


(16) $$\{\begin{array}{c} \int K(\mu )d\mu =1;\\ \int \mu K(\mu )d\mu =0;\\ \int \mu^{2}K(\mu )d\mu =\mu^{2}(k) \end{array}.$$

Gauss kernel was used to estimate the dynamic change of CCD between urbanization and flood disasters subsystems.


(17) $$K(\mu )=\frac{1}{\sqrt{2\pi }}e^{\frac{\mu^{2}}{2}}.$$

### System contribution rate

The system contribution rate is calculated as follows:


(18) $$C_{i}=\frac{X_{i}}{X_{U}+X_{R}+X_{S}},$$

where, $C_{i}$ is the contribution rate of system *i* (urbanization, flood resilience, or SOVI). $X_{U}$, $X_{R}$, and $X_{S}$ are the normalized values of urbanization (*U*), flood resilience (*R*), and SOVI (*S*). $X_{i}$ represents the value of the corresponding system.

Thus, for each subsystem:


(19) $$C_{U}=\frac{X_{U}}{X_{U}+X_{R}+X_{S}}$$

(20) $$C_{R}=\frac{X_{R}}{X_{U}+X_{R}+X_{S}}$$

(21) $$C_{S}=\frac{X_{S}}{X_{U}+X_{R}+X_{S}}.$$

By calculation, we can obtain the contribution rates of three systems in each province for each year. Comparing the contribution rate values of each system, we believe that the system with the highest value is the dominant system.

## Supplementary Material

pgag240_Supplementary_Data

## Data Availability

The data underlying this article are available in the article and in its online supplementary material. The raw data come from statistical yearbooks of various government departments in China: National Bureau of Statistics—*China Statistical Yearbook*: https://www.stats.gov.cn/sj/ndsj/. Ministry of Water Resources of the People's Republic of China—*China Water and Drought Disaster Bulletin*: http://www.mwr.gov.cn/sj/tjgb/zgshzhgb/. Ministry of Housing and Urban Rural Development of the People's Republic of China—*Statistical Yearbook of Urban and Rural Construction*: https://www.mohurd.gov.cn/gongkai/fdzdgknr/sjfb/tjxx/jstjnj/index.html. National Health Commission of the People's Republic of China-*China Health Statistics Yearbook*: http://www.nhc.gov.cn/mohwsbwstjxxzx/tjzxtjsj/tjsj_list.shtml. Because of potential data accessibility restrictions, all data and codes used in this study are available on Zenodo at DOI: 10.5281/zenodo.18253894. https://zenodo.org/records/18253895.
