# Supplementary material for: Tipping points in urban growth: How urbanization reshapes flood resilience and social vulnerability in China
Source: PNAS Nexus. 2026 Jul 9;5(7):pgag240. doi: 10.1093/pnasnexus/pgag240 (PMC13394701; doi:10.1093/pnasnexus/pgag240)
Supplement: pgag240_Supplementary_Data [file pgag240_supplementary_data.pdf]

Supplementary Information for
Tipping Points in Urban Growth: How Urbanization Reshapes Flood Resilience and Social Vulnerability in China

Xiguang Liu[1], Yue Wu[1], Rui Ren[1], Hussam N. Mahmoud[2,*]
[1]Institute for Resilient and Engineered Urban Planning, Institute for Interdisciplinary Innovation Research, Xi'an University of Architecture and Technology, Xi'an, Shaanxi, China
[2] Vanderbilt Center for Sustainability, Energy and Climate, Department of Civil and Environmental Engineering, Vanderbilt University, Nashville, TN, USA

* Hussam N. Mahmoud
Email: Hussam.Mahmoud@Vanderbilt.edu

This PDF file includes:

Supplementary text
Figures S1 to S13
Tables S1 to S9
Legends for Datasets S1
SI References

Other supplementary materials for this manuscript include the following:

Datasets S1

Supplementary Information Text

Additional Information on Methods

An indicator system was first established to evaluate the three dimensions of urbanization, flood resilience, and social vulnerability **(see more information in Tables S1-S3)**, and the raw data were standardized accordingly. A projection pursuit model, optimized using a genetic acceleration algorithm, was then applied to calculate the developments in the three systems **(see more information in Tables S4-S6)**. To explore the evolutionary characteristics of urbanization, flood resilience, and social vulnerability across regions and over time – and to identify possible mismatches or misalignment - we performed a spatiotemporal analysis of each province's development level. This includes the use of heat maps and spatial distribution maps **(see more information in Figures S2-S4)** to highlight regional disparities and reveal underlying spatial patterns across the three dimensions of development.

We conducted pairwise interaction analysis on the urbanization level, flood resilience level, and social vulnerability level calculated based on the indicator system **(see more information in Figures S5-S7)**. After confirming the significant correlation between the three domains, we developed and applied a ternary coupling coordination (CCD) model **(see method)** to quantify the degree of alignment among the three. This model enables us to assess whether each province achieved balanced alignment among urbanization, flood resilience, and social vulnerability, or whether disparities persisted. In this study, higher CCD values indicate more synchronized, mutually reinforcing change across urbanization, flood resilience, and social vulnerability, whereas lower values indicate misalignment or imbalance. Using the CCD results from 2006 to 2020, we analyzed temporal trends and spatial patterns **(see more information in Table S1 and Figures S8–S9)**. Furthermore, probability density functions and type proportion analyses were used to depict the evolution trajectories of provinces with different coordination types.

Recognizing that spatial clustering often reflects differences in regional governance capacity and development heterogeneity, we applied local spatial autocorrelation analysis to the CCD results using local Moran's **I (see Figure S10)**. This approach enabled the identification of four spatial clustering patterns - high–high, low–low, high–low, and low–high **(see more information in Figure S11)** - thereby uncovering regional disparities in coordinated development across regions and their potential spatial interdependencies. These results provide a basis for formulating differentiated governance approaches and fostering collaborative development strategies. Furthermore, to identify the key drivers that lead to coordinated outcomes in different periods and regions, we calculated the contribution rates of urbanization, flood resilience, and social vulnerability, and defined the dominant subsystem types at each time point **(see Figure S13 and Table S3)**. This analysis helps clarify the specific sources of regional differences in coordinated development, whether they stem from uneven urbanization, insufficient flood resilience, or high social vulnerability.

Background information on flood disasters in China from 2006 to 2020

This section introduces the impact of floods disasters on China in recent years. China is also deeply mired in the dilemma of global climate extremes (1).

Fig. S1 displays the specific annual values of flood-related losses in China over a 15-year period. The peaks in various types of losses correspond to the same year, reflecting the severity of the flood disasters in that year. Overall, between 2006 and 2020, the direct economic losses due to floods in China totaled approximately 3 trillion RMB. These floods resulted in the collapse of 4.07 million houses and affected 140 million hectares of arable land. The total number of people affected by flood disasters during this period was around 1.6 billion.

Additional data used to evaluate the level of urbanization, flood resilience and social vulnerability

This section provides all the details of the supplementary data used in our analysis to evaluate the level of urbanization, flood resilience and social vulnerability (2,3). These data elements are crucial for understanding the urbanization level of various provinces in China and their changes in urban resilience in the face of floods disasters.

Table S1-S3 displays the types of indices included in the basic dataset, as well as the impact of each index on different systems. The indicators of urbanization system are divided into five categories, including population, economy, land use, social and livelihood urbanization. The flood resilience system includes three stages: pre disaster, during disaster, and post disaster. Pre-disaster indices address the city's preparedness prior to disaster occurrences. During-disaster indexes include rainfall intensity, the frequency of flood events in urban areas, and the stability of communication systems during disasters. Post disaster indexes include indexes of the impact of post disaster reconstruction and indexes reflecting the city's recovery capacity. Social vulnerability includes five types: population, healthcare, education, infrastructure, and socio-economic status Positive indexes are labeled as TRUE, while negative indexes are labeled as FALSE, which affects the process of data normalization.

Datasets S1 contains all the data used in this study. This file contains all the variables data used in 31 provinces of China from 2006 to 2020. Each year's data is stored separately in a sheet for easy access. For some missing statistical data, we used linear interpolation to supplement them.

Evaluation results of urbanization system and urban flood resilience system

This section presents all the results of China's urbanization level evaluation, flood resilience and SOVI evaluation. It contains the specific numerical values we have calculated, as well as maps that spatially connect these values with various provinces in China.

Table S4 demonstrates the urbanization level score calculated using a projection pursuit evaluation model based on genetic acceleration algorithm2. Due to the randomness of genetic acceleration algorithm, we selected a more suitable set of data from the various results obtained.

Table S5 demonstrates the flood resilience level score calculated using a projection pursuit evaluation model based on genetic acceleration algorithm2. Due to the randomness of genetic acceleration algorithm, we selected a more suitable set of data from the various results obtained.

Table S6 demonstrates the social vulnerability level score calculated using a projection pursuit evaluation model based on genetic acceleration algorithm. Due to the randomness of genetic acceleration algorithm, we selected a more suitable set of data from the various results obtained.

Fig. S2 shows the spatial distribution of the calculated urbanization level values in China. Overall, China's urbanization level has greatly improved between 2006 and 2020. The urbanization level in the northwest inland region of China lags slightly behind that in the southeast coastal region. It is normal for the urbanization level values of some provinces to fluctuate in certain years, such as Tibet. This may be due to the lack of relevant statistical data for that year and the use of linear interpolation to supplement the data, or it may be due to errors in government statistical data for that year.

Fig. S3 shows the spatial distribution of China's flood resilience level values obtained through calculation. Overall, the resilience level of urban floods in China has been improved between 2006 and 2020. The most significant improvement in urban flood resilience is observed in the southeastern coastal regions of China. This is because they have a higher frequency of flood disasters. The local government actively takes measures to cope with frequent flood disasters.

Fig. S4 shows the spatial distribution of China's SOVI level values obtained through calculation. From 2006 to 2020, the overall level of social vulnerability in China showed a downward trend, with SOVI values decreasing year by year. It indicates that social resilience is constantly increasing in most regions. The decline in SOVI is particularly evident in the eastern and some central provinces, while although there has been some improvement in the western region, the overall vulnerability is still high, and regional differences still exist over time.

Linear correlation between urbanization, urban flood resilience and social vulnerability

Fig. S5 presents the linear regression analysis between the comprehensive level of urbanization and flood resilience in 31 provinces of China from 2006 to 2020. Each subplot represents a single province, displaying the scatter points of annual data, the fitted linear regression line, and the 95% confidence interval (shaded area). The coefficient of determination (R^2) is labeled in each panel, indicating the goodness-of-fit of the regression model. Most provinces show a strong positive correlation between

urbanization and flood resilience ($R^2 > 0.90$), suggesting that urban development has generally contributed to enhanced infrastructure resilience. However, a few regions such as Beijing and Tibet exhibit relatively weaker correlations ($R^2 < 0.70$), implying that in these areas, resilience improvement may not be solely driven by urbanization. The variation in slope and fit across regions reflects the heterogeneity of development strategies and flood management capacities in different parts of China.

Fig. S6 shows the linear regression between the comprehensive level of urbanization and Social Vulnerability (SOVI) in 31 provinces of China from 2006 to 2020. Each subplot represents a single province, displaying the scatter points of annual data, the fitted linear regression line, and the 95% confidence interval (shaded area). The coefficient of determination (R^2) is labeled in each panel, indicating the goodness-of-fit of the regression model. Most provinces show a strong negative correlation ($R^2>0.90$), suggesting that higher urbanization levels are generally linked with lower social vulnerability. This indicates that people in more urbanized areas tend to have a stronger ability to cope with flood risks. However, in some provinces such as Tibet and Hainan, the correlation is weaker, which may be due to local social, economic, or geographic factors. The differences in regression results across regions highlight the spatial variation in how urbanization affects social vulnerability in China.

Fig. S7 presents the linear regression analysis between the comprehensive level of SOVI and flood resilience in 31 provinces of China from 2006 to 2020. Each subplot represents a single province, displaying the scatter points of annual data, the fitted linear regression line, and the 95% confidence interval (shaded area). The coefficient of determination (R^2) is labeled in each panel, indicating the goodness-of-fit of the regression model. In most provinces, a strong negative relationship is observed ($R^2>0.90$), suggesting that higher flood resilience is generally associated with lower social vulnerability. This means that improvements in infrastructure and disaster preparedness can help reduce the vulnerability of local populations. However, some provinces such as Tibet and Shaanxi show weaker correlations, possibly due to regional differences in development conditions or disaster exposure. The results highlight spatial variations in the interaction between resilience and social vulnerability in China.

Evaluation results of coupling coordination degree level

This section presents all the results of coupling coordination degree level. It contains the specific numerical values we have calculated, as well as maps that spatially connect these values with various provinces in China. We classify the coupling coordination relationship and conduct grouping analysis on provinces.

Table S7 demonstrated the coupling coordination degree (CCD) values calculated using an improved coupled coordination model. The CCD value represents the coupling and coordination relationship between urbanization system, flood resilience and SOVI systems.

Table S8 demonstrates the division of CCD values (4,5). Dividing CCD values can better help us understand the degree of coupling and coordination between urbanization systems and urban flood resilience systems in various provinces, and group them accordingly.

Fig. S8 shows the spatial distribution of the calculated CCD values. Overall, the CCD values of various provinces in China have increased relatively slowly between 2006 and 2020, and are generally low. The provinces in the southeastern coastal region of China have undergone significant changes.

Fig. S9 illustrates the annual spatial distribution of the CCD among urbanization, flood resilience, and social vulnerability across 31 provinces in China from 2006 to 2020. The provinces are categorized into six CCD levels, with warmer colors indicating higher coordination. Over time, the CCD level improves significantly in eastern and coastal regions, while central and western areas show slower progress.

Global spatial autocorrelation report of CCD values in 31 provinces of China.

This section presents a report on global spatial autocorrelation.

Fig.S10 was generated by ArcGIS and shows a summary of the global Moran's I index, used to evaluate the autocorrelation of spatial data, i.e. whether spatially close data points tend to exhibit similar values. Moran's I index is used to measure spatial autocorrelation. The range of values is usually between -1 and 1, with positive values indicating positive spatial autocorrelation, meaning that spatially close positions tend to have similar values. The spatial distribution of CCD values in China has a positive spatial correlation.

Expected index represents the expected Moran's I index under random conditions. The actual calculated Moran's I value is far from this value, indicating that the spatial mode of CCD is non-random. Variance is the variance of Moran's I index, used to calculate Z-score and P-value, and further determine statistical significance. Z-score represents the number of standard deviations between Moran's I index and its expected value. The Z-score value is 2.836, indicating that Moran's I index is higher than its random expected value, and this bias is statistically significant.

P-value is used to test the statistical significance of Moran's I index. Here, the P-value is 0.004565, which means there is a very small probability (less than 1%) that Moran's I index is generated by a random process.

Fig. S11 shows the spatial clustering analysis of the ternary Coupling Coordination Degree (CCD) among urbanization, flood resilience, and SOVI in China from 2006 to 2020 reveals significant regional disparities and evolving spatial dependencies. Provinces divides into four categories—H-H (High-High), L-L (Low-Low), H-L (High-Low), and L-H (Low-High). It illustrates the spatial clustering of high and low coordination levels across different regions. Over time, a general trend toward improved coordination is observed, but certain regions continue to exhibit persistent challenges.

Between 2006 and 2010, H-H clusters were concentrated in highly urbanized and economically developed areas such as Beijing, Tianjin, and Shanghai. In contrast, L-L clusters were primarily found in provinces such as Shanxi, Guizhou, and Qinghai. It indicates that due to economic constraints and underdeveloped flood control strategies in these provinces, the overall development of the region lags behind other provinces. Other provinces such as Hebei and Inner Mongolia are classified as L-H or H-L. This indicates that their development is not coordinated with the surrounding areas.

Between 2011 and 2015, H-H clusters expanded, with Jiangsu, Zhejiang, and Guangdong joining the ranks of high CCD provinces. However, L-L clusters remained largely unchanged, particularly in western provinces. It highlights the continued difficulties in integrating urbanization and resilience planning in underdeveloped regions. A noticeable increase in H-L clusters, especially in areas adjacent to economically strong provinces. This indicates a widening CCD gap between some provinces and their surrounding regions.

Between 2016 and 2020, western provinces continued to exhibit L-L clustering. It reflects the entrenched nature of low CCD, with limited improvements over time. H-L and L-H classifications became more common in this period. This indicates more frequent CCD mismatches between provinces and their neighbors, and it also suggests less synchronized progress across urbanization, flood resilience, and SOVI.

Regionally, eastern coastal provinces such as Shanghai, Jiangsu and Zhejiang have consistently exhibited high CCD, with stable H-H clustering patterns. However, Beijing and Tianjin stay H-H through 2018. Both switch to L-L in 2019. In 2020, Beijing becomes H-L, while Tianjin becomes L-H. These shifts indicate that Beijing and Tianjin moved from a stable H-H clustering pattern to a brief L-L phase in 2019, followed by a divergence in 2020 that reflects weaker spatial synchrony with neighboring provinces. In central provinces such as Henan, Hunan, and Hubei, clustering patterns indicate ongoing transitions, but the trajectories differ across provinces. Henan shifts from L-L in 2006–2011 to L-H from 2012 onward. Hunan remains L-L through 2016 and turns to H-L during 2017–2020. Hubei is predominantly classified as H-L across the period, with brief H-H years in 2017 and 2019. In contrast, several western provinces, including Tibet, Guizhou, and Qinghai, remain persistently in L-L clusters throughout 2006–2020. Inner Mongolia is consistently classified as H-L, indicating a long-term high–low mismatch with surrounding areas. In the northeast, Heilongjiang transitions from H-H during 2006–2016 to L-L during 2017–2020, suggesting a notable weakening of spatial clustering over time.

Overall, spatial disparities in ternary CCD persist over 2006–2020.There is a stable high-coordination core in the east. Shanghai, Jiangsu, and Zhejiang remain H-H throughout the period. Several western provinces, including Tibet, Guizhou, and Qinghai, remain L-L in all years.

Mismatch types become more common in 2020. H-L increases from 5 in 2006 to 7 in 2020. L-H increases from 3 in 2006 to 6 in 2020. These patterns indicate that CCD improvements are not spatially synchronized across neighboring provinces.

Therefore, the future work and policy discussions should distinguish between persistently L-L regions and H-L/L-H mismatch regions. These two groups may require different intervention priorities. Cross regional coordination may need to be strengthened.

Specific analysis of changes in 31 provinces

Fig.S12 shows the evolution trend of the CCD system of urbanization, flood resilience, and SOVI in 31 provinces of China from 2006 to 2020. From 2006 to 2010, ternary CCD levels were generally lower, with clear regional heterogeneity. Several eastern coastal municipalities and provinces already showed relatively higher CCD, while many inland and western provinces remained at low levels.
During 2011–2015, CCD increased in a number of central and eastern provinces, indicating gradual improvement in overall coordination.

After 2016, more provinces moved into higher CCD levels, suggesting broader progress in coordinated development across urbanization, flood resilience, and SOVI.
However, some provinces continued to exhibit persistently low CCD, implying difficulties in achieving balanced improvement across the three components.

Fig. S13 shows the contribution proportion of urbanization, flood resilience, and SOVI in 31 provinces of China to the CCD from 2006 to 2020.
From 2006 to 2020, SOVI dominance decreases markedly over time. In 2006–2009, SOVI is dominant in 27 of the 30 provinces shown, whereas by 2020 SOVI remains dominant only in Yunnan, Tibet, and Gansu. In contrast, urbanization becomes dominant in an increasing number of provinces, including Beijing, Tianjin, and Shanghai throughout 2006–2020. Flood resilience also becomes dominant in more provinces over time and is dominant in 2020 in Hebei, Jiangsu, Shandong, Henan, Guangdong, and Sichuan. Overall, the results indicate a broad shift in dominant contributions from SOVI toward urbanization or flood resilience in many provinces, but a few western provinces remain SOVI-dominant.

Table S9 shows from 2006 to 2020, the CCD level of various provinces in China showed an overall upward trend, but there were significant regional differences. Meanwhile, there are significant differences in the types of dominant systems among different provinces, with urbanization systems being the most common. It indicates that urban development plays a core role in the coordination of the three systems. In addition, the temporal changes of clustering types also show that some provinces have gradually shifted from low-level combinations to high-level coordination patterns. This reflects the gradual optimization of the system interaction mechanism and the obvious trend of regional coordinated development.

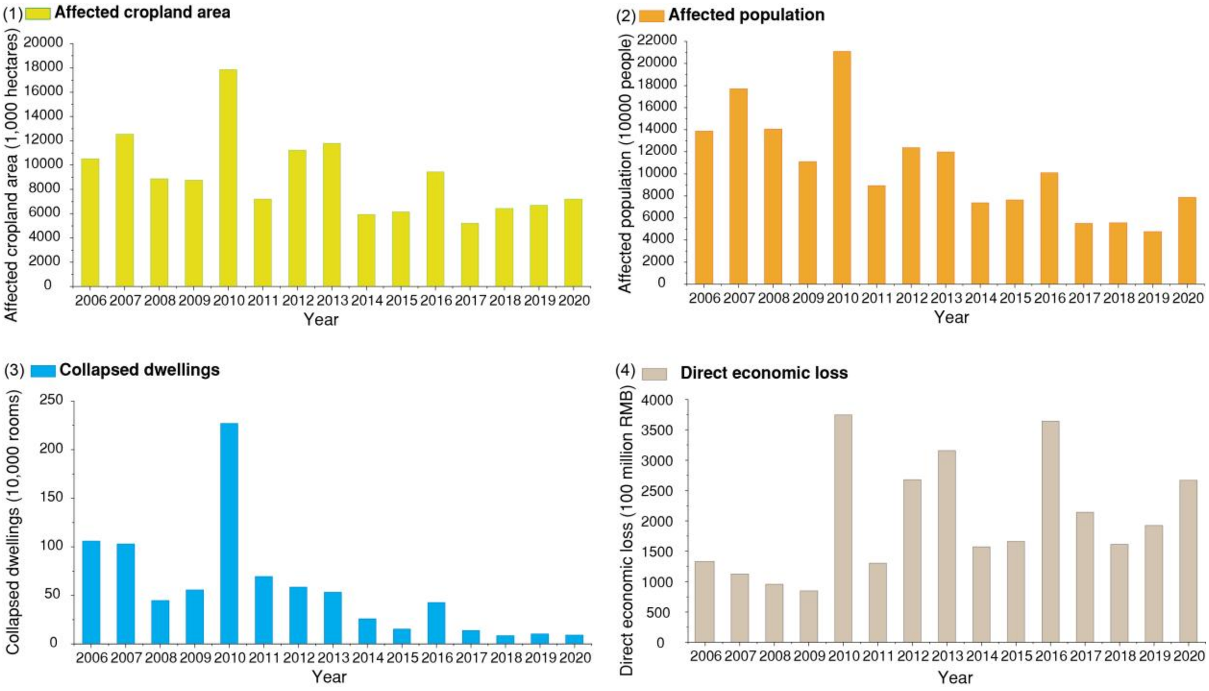

Fig. S1. Statistics of Flood Disasters in China from 2006 to 2020.

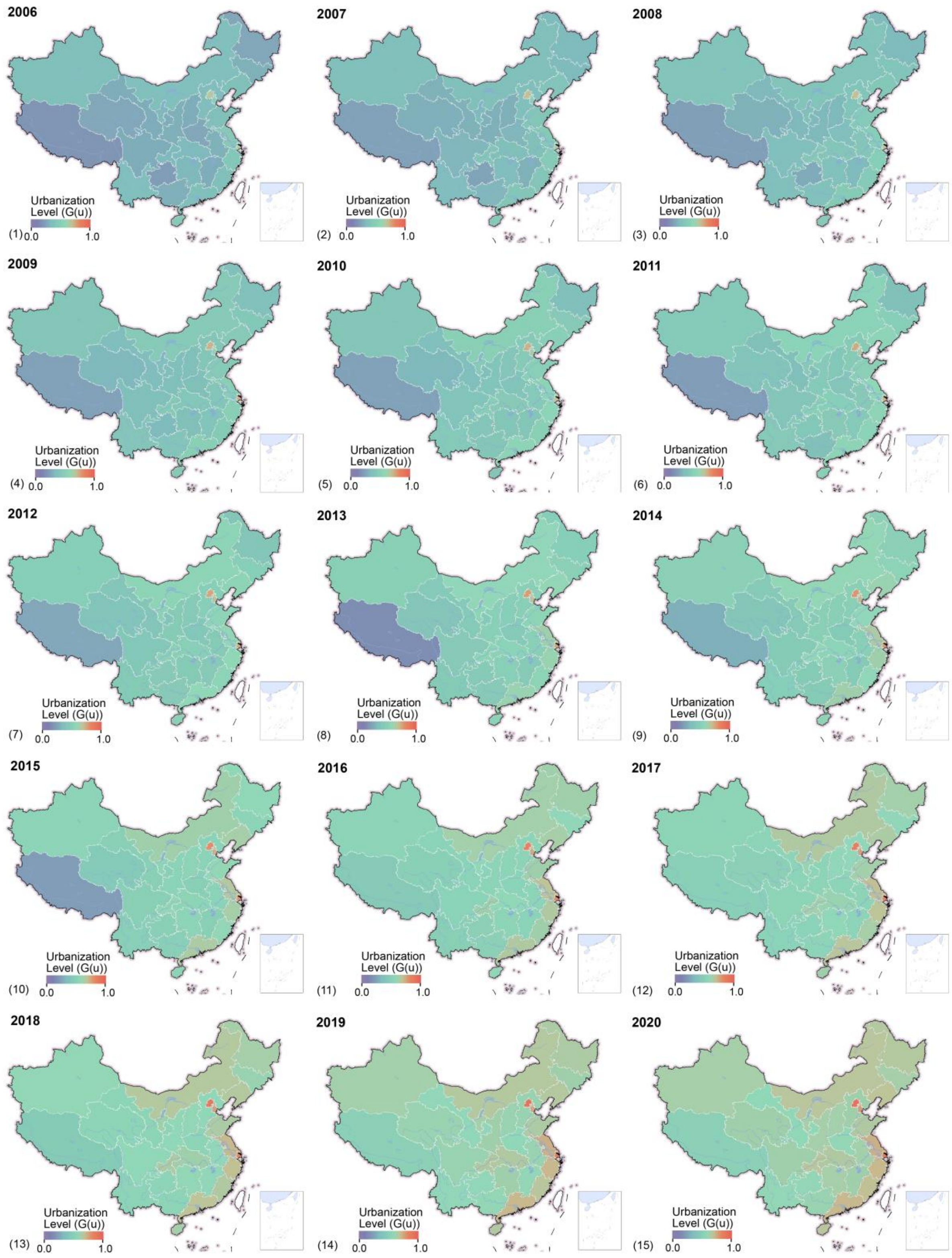

Fig. S2. The spatial distribution changes of urbanization level in China.

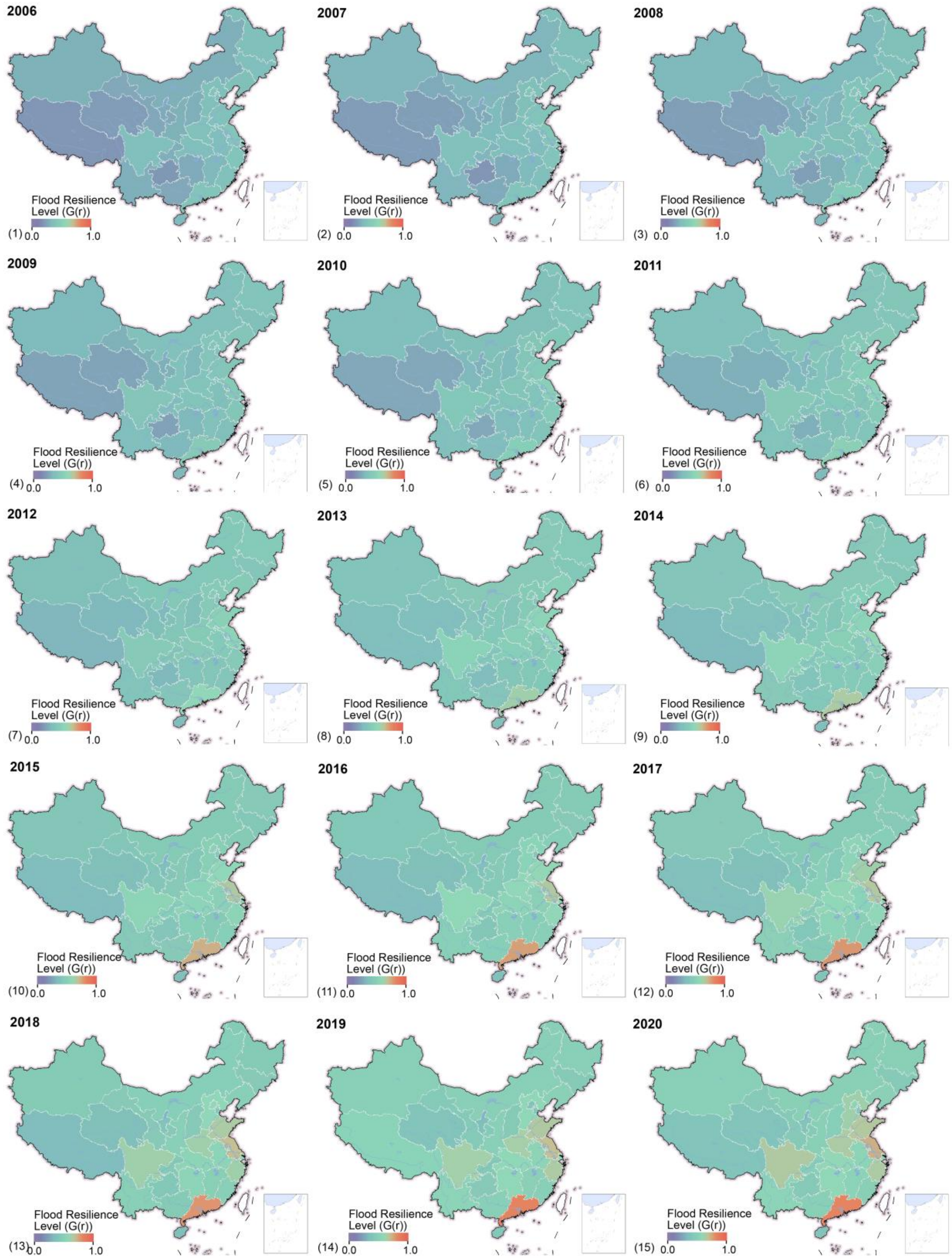

Fig. S3. The spatial distribution changes of resilience level in China.

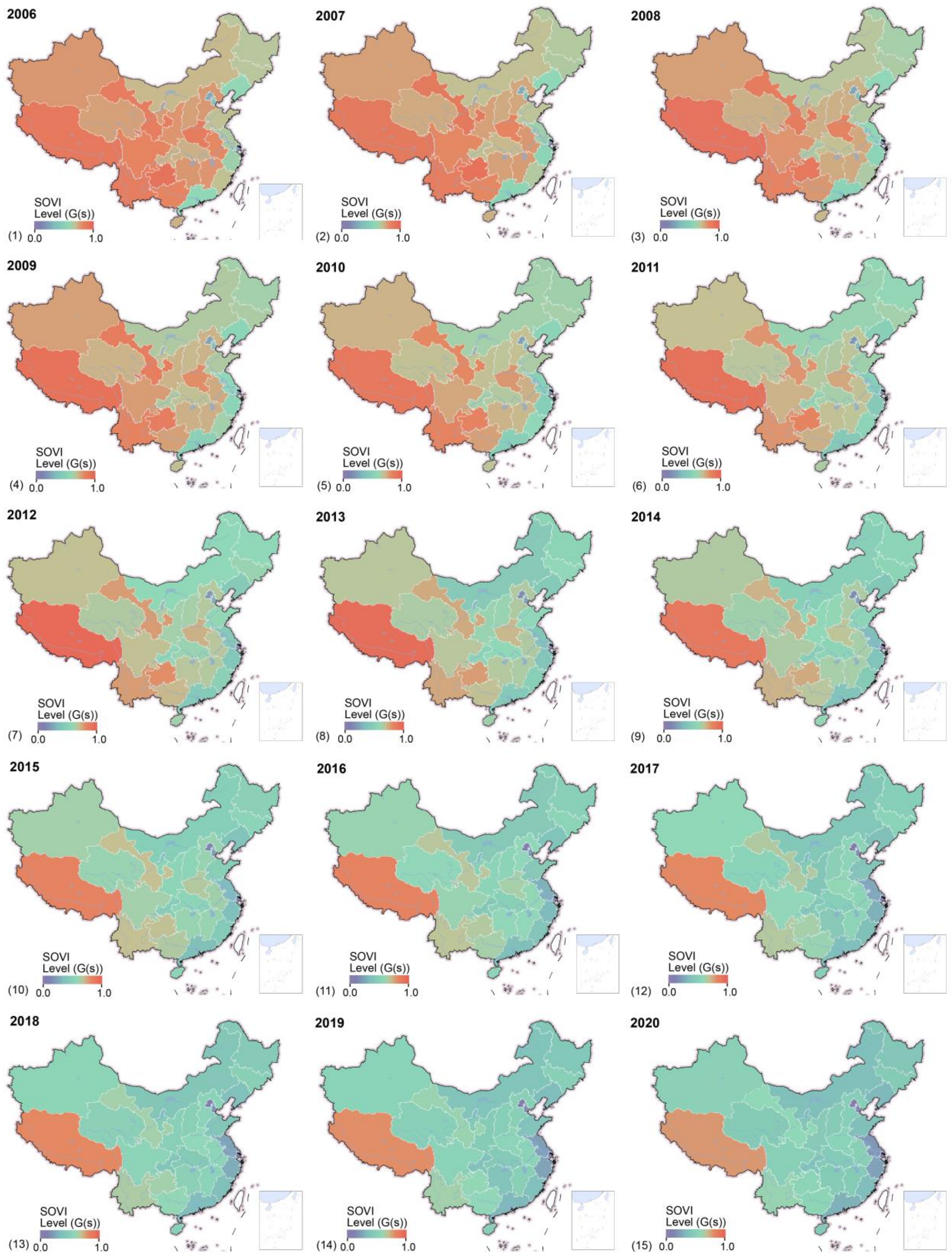

Fig. S4. The spatial distribution changes of SOVI level in China.

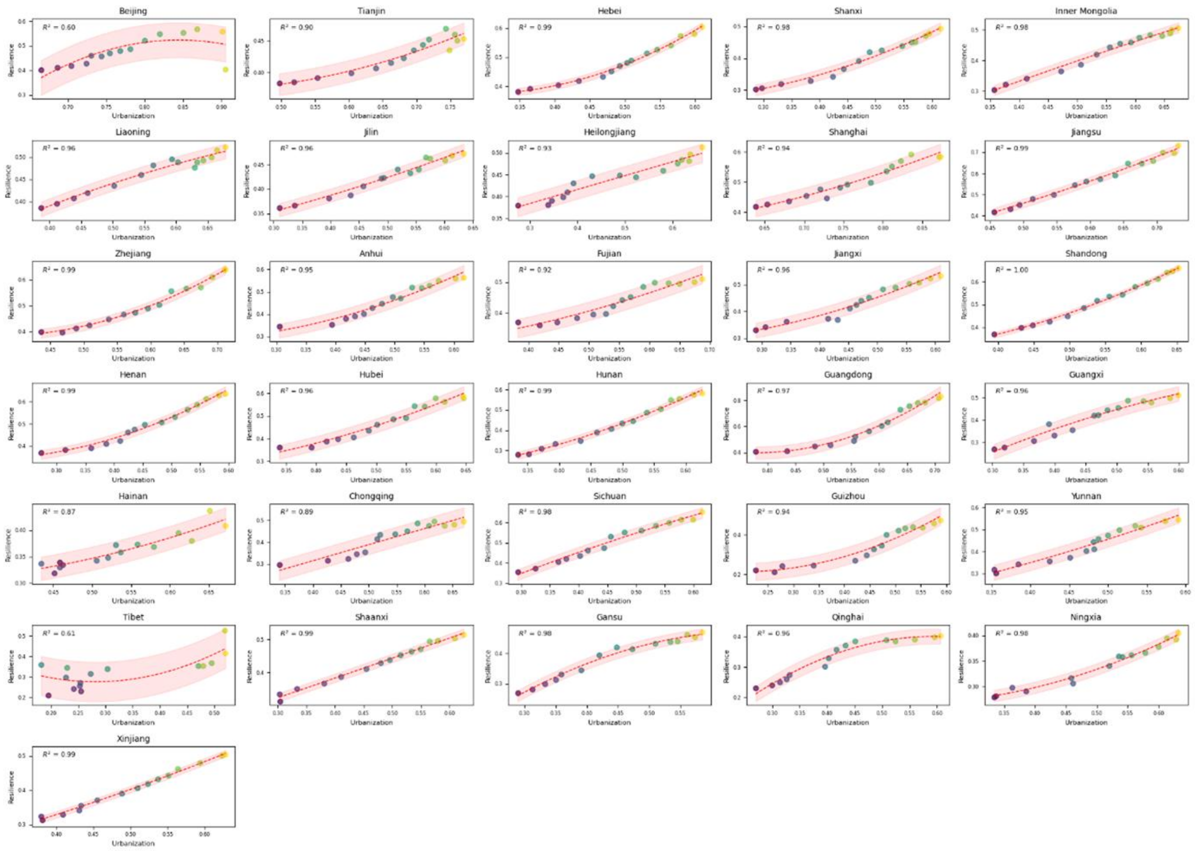

Fig. S5. Linear fitting of urbanization and flood resilience in 31 provinces of China.

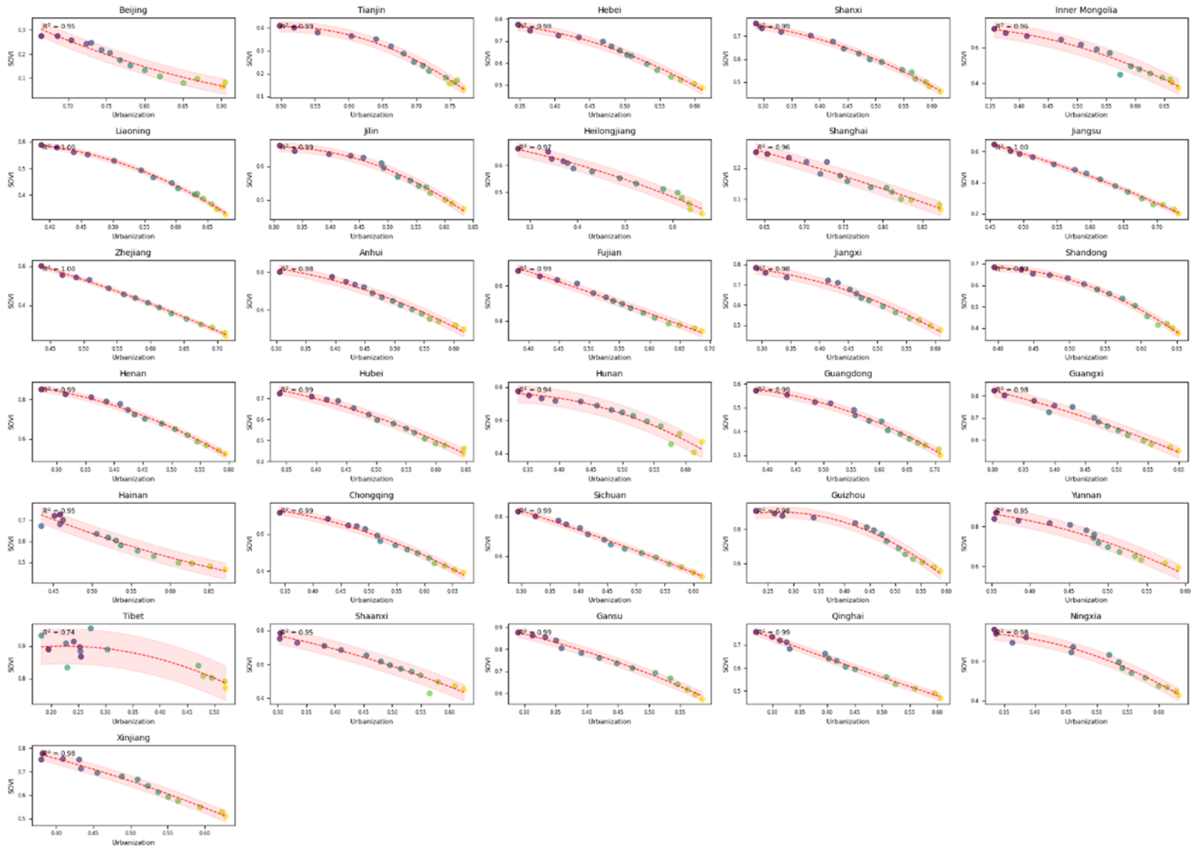

Fig. S6. Linear fitting of urbanization and SOVI in 31 provinces of China.

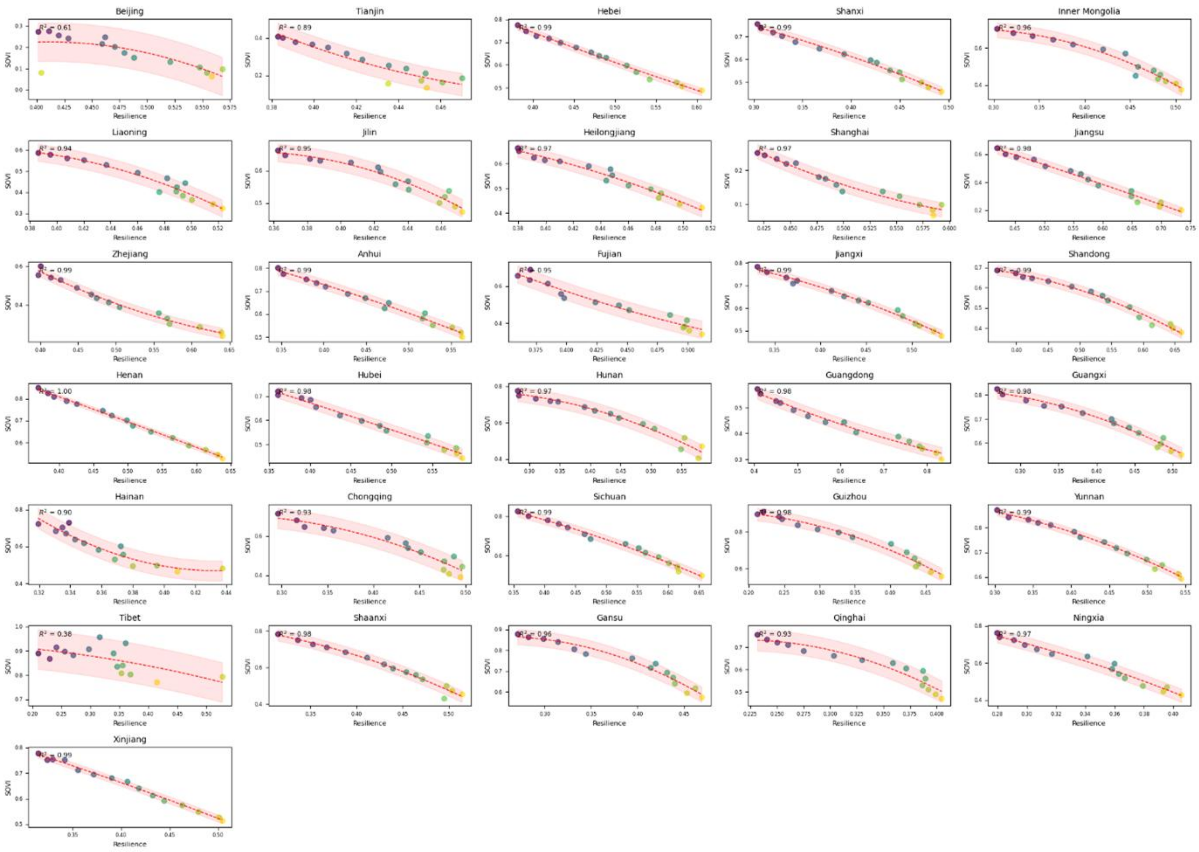

Fig. S7. Linear fitting of SOVI and flood resilience in 31 provinces of China.

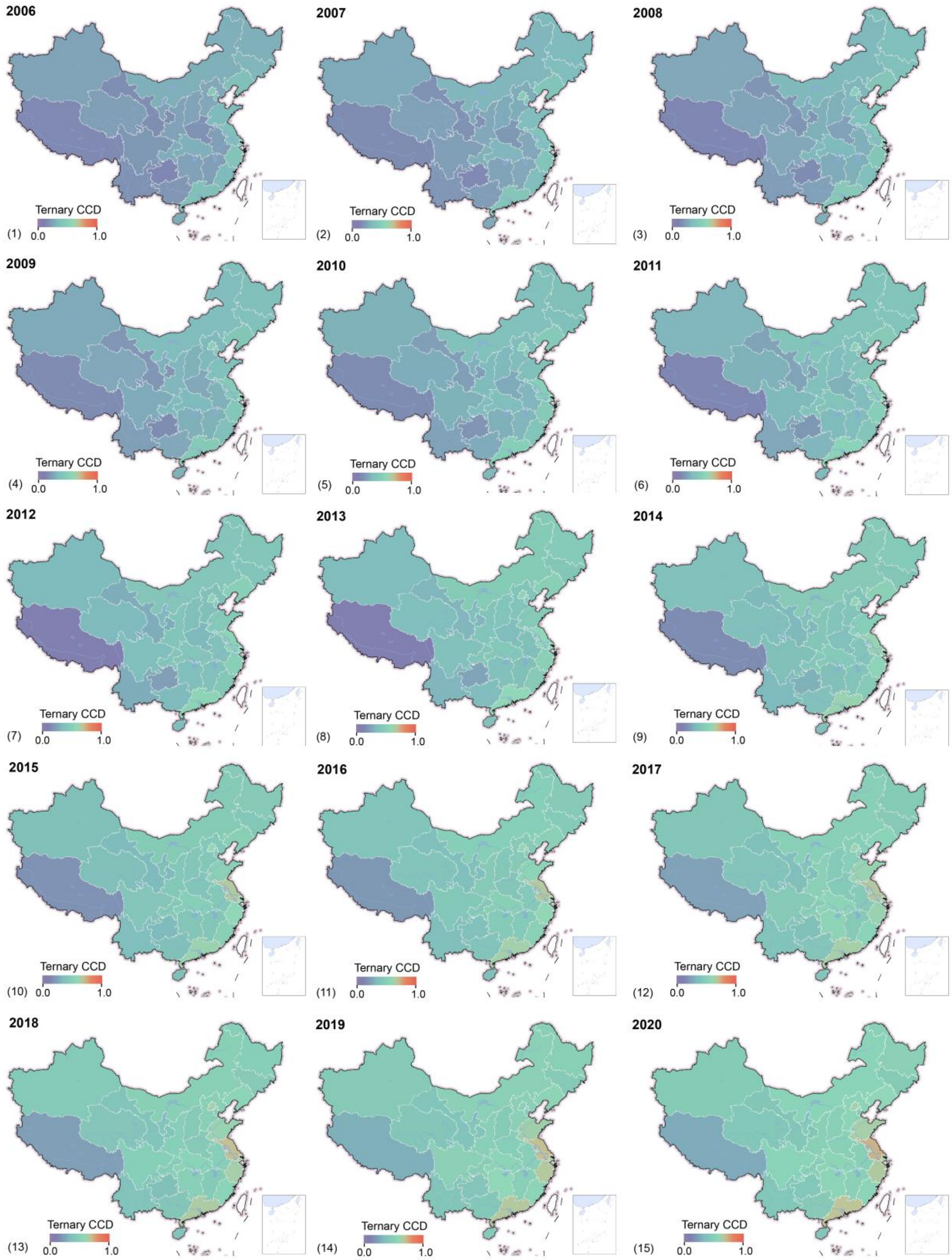

Fig. S8. Changes in the Ternary CCD Values of 31 Provinces from 2006 to 2020.

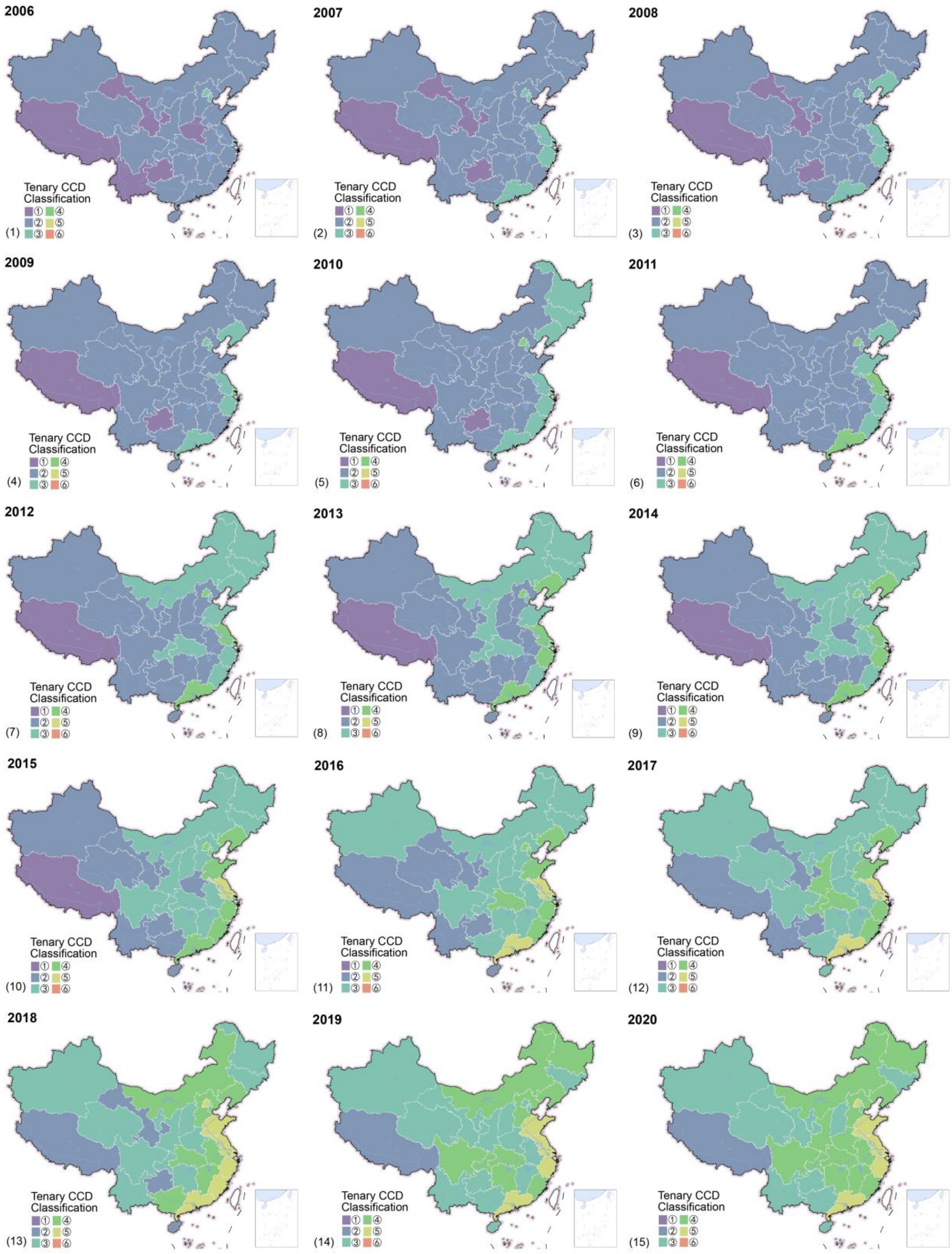

Fig. S9. Changes in the Ternary CCD Classification of 31 Provinces from 2006 to 2020.

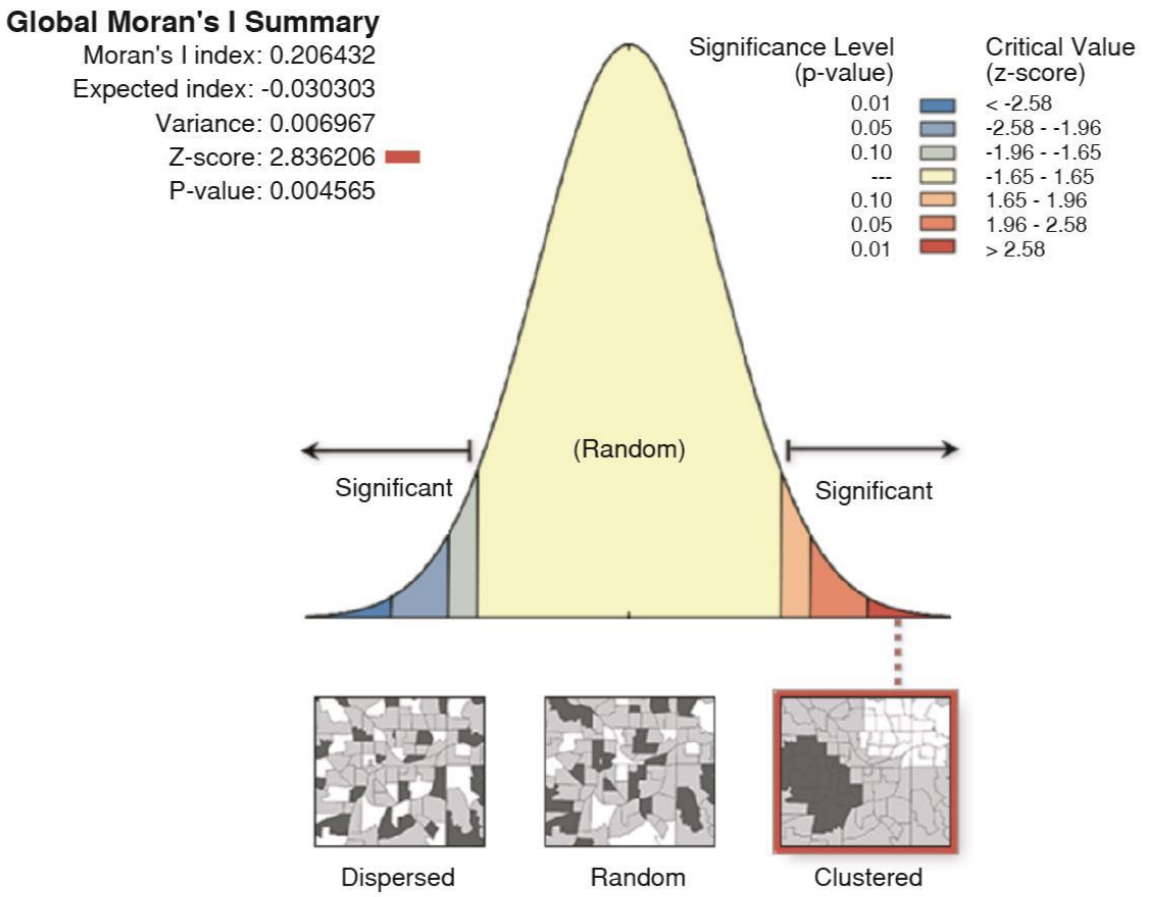

The z-score of 2.83620559539 indicates that the probability of randomly generating this clustering pattern is less than 1%.

Fig. S10. Global spatial autocorrelation report of CCD values in 31 provinces of China.

a

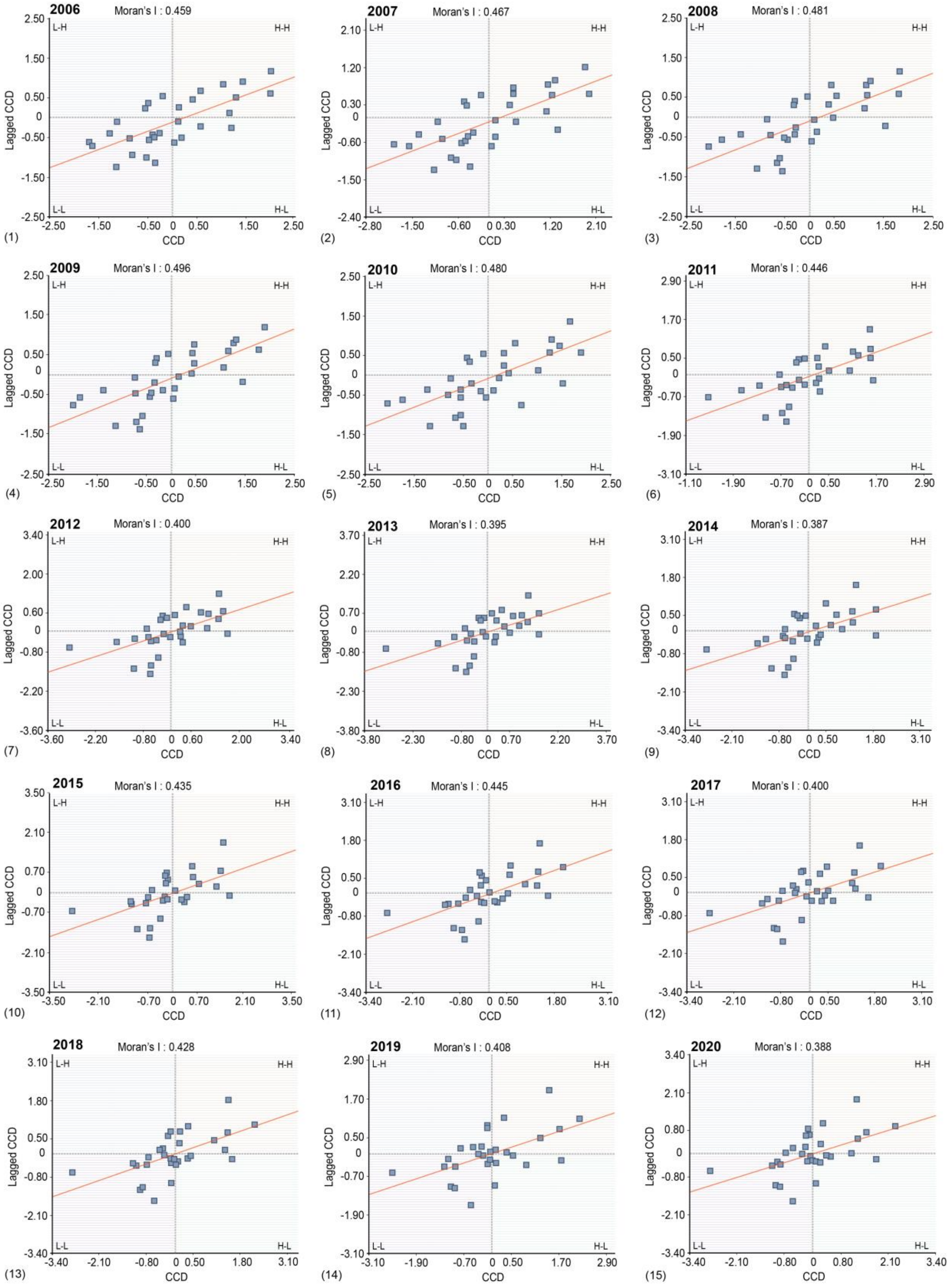

Fig. S11. Local spatial autocorrelation analysis and clustering characteristics of 30 provinces in China from 2006 to 2020.

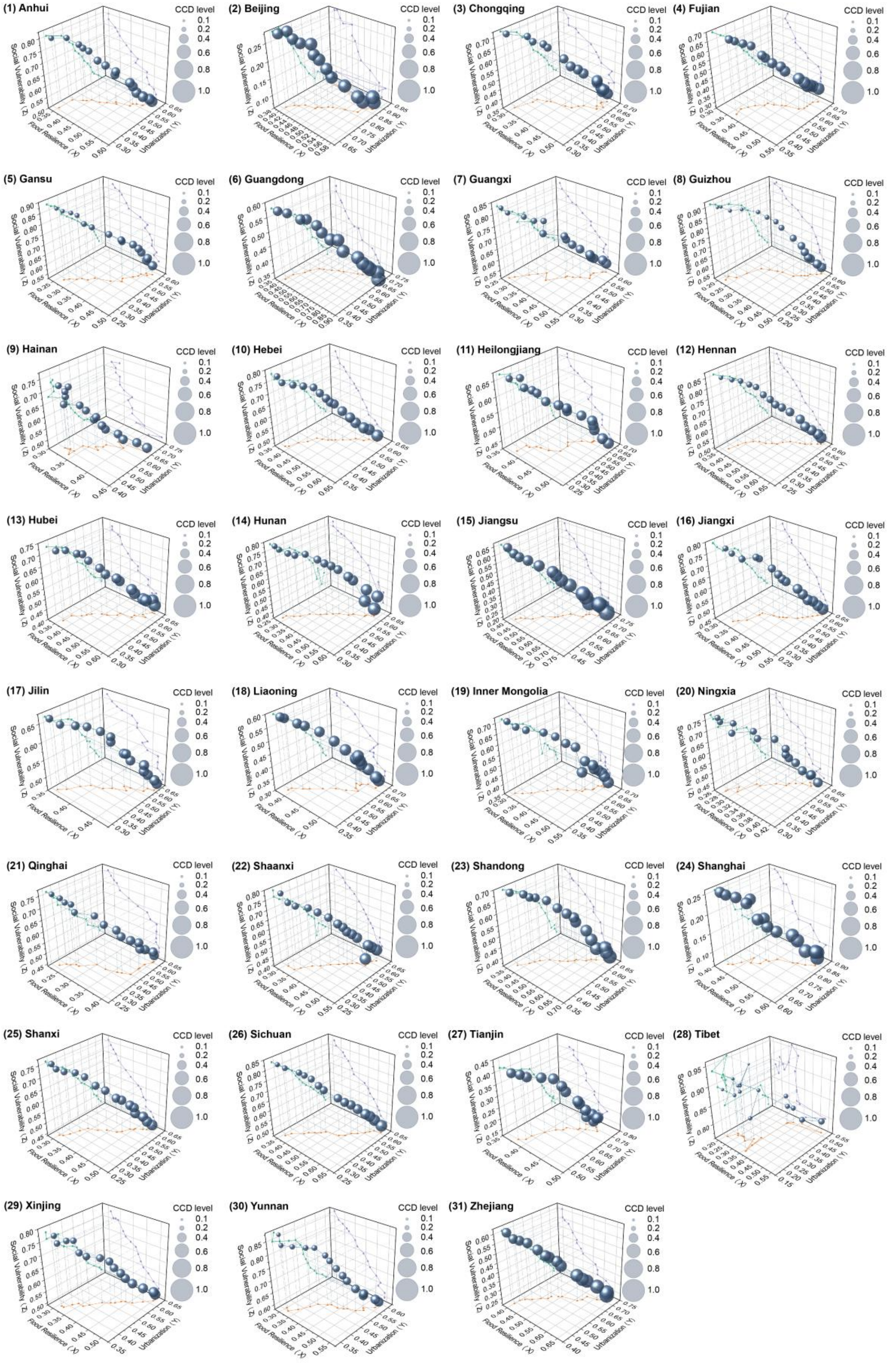

Fig. S12. Changes in the Ternary CCD Classification of 31 Provinces from 2006 to 2020.

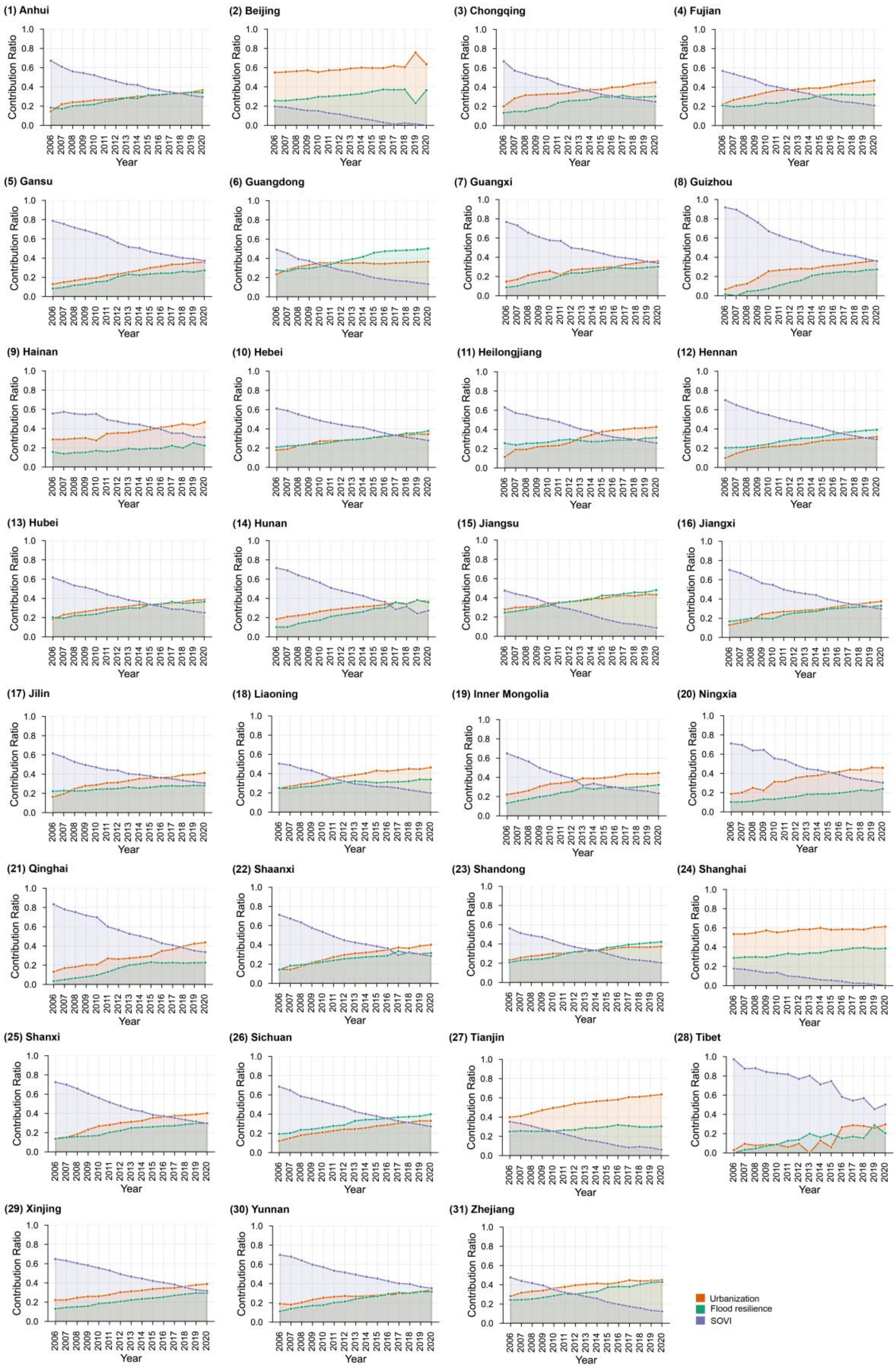

Fig. S13. Changes in contribution rates of the three systems in each province.

Table S1. Urbanization System Indices.

System	Content	Index name	Variables (Unit）	Index property
Urbanization system	Demographic Urbanization	Population	Urban population percentage (%)	TRUE
			Population density (person/km^2)	TRUE
			The proportion of the population in the tertiary industry to the employed population (%)	TRUE
	Economic development	Economic	Per capita GDP (yuan)	TRUE
			Per capita consumption expenditure of urban residents (yuan)	TRUE
			The proportion of the second industries to GDP (%)	FALSE
			The proportion of the third industries to GDP (%)	TRUE
	Spatial urbanization	Land use	Per capita area of urban roads (m^2)	TRUE
			Urban construction land area (km^2)	TRUE
			Urban per capita residential land area (km^2)	FALSE
			Green space ratio in built-up areas (%)	TRUE
	Urbanization of society	Society	Number of beds in medical and health institutions per thousand people(number)	TRUE
			Number of college students (person)	TRUE
			Urban unemployment rate (%)	FALSE
	Urbanization of life	Life	Water supply penetration rate (%)	TRUE
			Domestic waste treatment rate (%)	TRUE
			Sewage treatment rate (%)	TRUE

Definitions of Urbanization System Indices:

Population Index
The Population Index measures the demographic dimension of urbanization, reflecting the concentration of population, the degree of population agglomeration, and the transition of labor toward urban economic activities. Higher values indicate a greater level of demographic urbanization.

Economic Index
The Economic Index measures the level of economic development and structural transformation associated with urbanization. It reflects economic productivity, household welfare, consumption capacity, and the shift toward service-oriented economic activities. Higher values indicate a more advanced stage of economic urbanization.

Land Use Index
The Land Use Index measures the spatial expansion and physical development of urban areas. It reflects the intensity of land development, transportation infrastructure, residential land utilization, and the provision of urban green spaces. Higher values indicate a higher degree of spatial urbanization and urban land development.

Society Index
The Society Index measures the social dimension of urbanization, including access to education, healthcare resources, and employment opportunities. It reflects the capacity of urban areas to provide social services and support human development. Higher values indicate a higher level of social urbanization.

Life Index
The Life Index measures the quality and accessibility of basic urban services that support residents' daily living conditions. It reflects the provision of essential municipal services, including water supply, waste management, and wastewater treatment. Higher values indicate a higher quality of urban living and service provision.

Table S2. Flood Resilience System Indices.

System	Content	Index name	Variables (Unit）	Index property
Flood resilience system	Before the disaster	Disaster prevention measures	General public budget expenditure (10000 yuan)	TRUE
			Number of reservoirs (number)	TRUE
	During disasters	Disaster intensity	Annual precipitation (mm)	FALSE
			Number of flood events (number)	TRUE
		Stability of communication system	Coverage rate of the event on TV (%)	TRUE
			Coverage rate of the event on broadcast (%)	TRUE
	Post disaster	Post disaster reconstruction	Total output value of construction industry (100 million yuan)	FALSE
			Flood control area (hectares)	FALSE
		Recovery level	Cropland restoration rate (%)	TRUE
			Affected population (10000 people)	FALSE
			Main agricultural product yield per unit area (kg/ha)	TRUE

Definitions of Flood Resilience System Indices:

Disaster Prevention Measures (Pre-disaster Capacity)
This index reflects the capacity of a region to prevent, mitigate, and prepare for flood hazards before they occur. It is characterized by indicators such as public budget expenditures on disaster prevention and the availability of water-control infrastructure (e.g., reservoirs). Higher values indicate stronger preparedness and greater capacity to reduce flood impacts.

Disaster Intensity (Hazard Exposure)
This index captures the magnitude and frequency of flood-related hazards experienced by a region. It is represented by annual precipitation and the number of flood events. Higher values indicate greater exposure to flood hazards and increased pressure on the resilience system.

Stability of the Communication System (Information and Warning Capacity)
This index measures the effectiveness and reliability of information dissemination during flood events. Indicators such as television and broadcast coverage rates reflect the ability of authorities to communicate warnings, emergency information, and response guidance to the public. Higher values indicate stronger communication capacity and improved emergency coordination.

Post-disaster Reconstruction Capacity
This index represents the ability of a region to restore infrastructure and essential services following flood events. It is measured through indicators such as the total output value of the construction industry and flood-control area. Higher values suggest a greater capacity for reconstruction and recovery.

Recovery Level (Post-disaster Recovery Performance)
This index reflects the extent to which socioeconomic and agricultural systems recover after a flood event. It is characterized by indicators such as cropland restoration rates, affected population, and agricultural productivity. Higher recovery levels indicate a stronger ability to restore normal functioning and reduce long-term flood impacts.

Table S3. Social Vulnerability System Indices.

System	Content	Index name	Index property
SOVI System	Consumption level	Rural consumption (yuan)	TRUE
		Urban consumption (yuan)	TRUE
	Unemployment	Urban unemployment rate (%)	FALSE
	Ethnicity	Ethnic minority ratio (%)	FALSE
	Age structure	Population aged 65+ ratio (%)	FALSE
		Population aged 0-14 ratio (%)	FALSE
	Urban population ratio	Urban population ratio (%)	TRUE
	Social security	Rural minimum living guarantee number(person)	FALSE
		Urban minimum living guarantee number(person)	FALSE
	Population growth	Natural population growth rate (%)	TRUE
		Female without education ratio (%)	FALSE
	Education	Illiteracy rate (15+) (%)	FALSE
	Healthcare	Health technicians per 1000 people(number)	TRUE
		Urban water supply penetration rate (%)	TRUE
	Infrastructure	Urban gas penetration rate (%)	TRUE
		Per capita generator capacity(kW/person)	TRUE
	Transportation	Public buses per 10,000 people(number)	TRUE

Definitions of Social Vulnerability System Indices

Consumption Level (Economic Capacity)
This dimension reflects the economic resources available to households and their ability to prepare for, withstand, and recover from disasters. It is represented by rural and urban consumption expenditures. Higher consumption levels generally indicate lower social vulnerability due to greater financial capacity and access to resources.

Unemployment
This dimension captures economic insecurity and limited access to stable income sources. The urban unemployment rate is used to characterize labor-market vulnerability. Higher unemployment levels are associated with greater social vulnerability.

Ethnicity
This dimension reflects potential disparities in socioeconomic opportunities, access to public services, and disaster resources among minority populations. The ethnic minority ratio is used to represent demographic groups that may face elevated vulnerability due to social and institutional inequalities.

Age Structure
This dimension represents demographic dependency and the presence of populations requiring additional assistance during disasters. The proportions of elderly individuals (65+) and children (0–14 years) are used as indicators because these groups often have reduced mobility and greater recovery needs.

Urban Population Ratio
This dimension captures the degree of urbanization and population concentration. A higher urban population ratio may indicate greater access to infrastructure, public services, and emergency resources, thereby reducing social vulnerability.

Social Security
This dimension reflects the extent of socioeconomic disadvantage and dependence on public assistance programs. The numbers of rural and urban minimum living guarantee recipients are used to characterize populations with limited financial resilience and greater susceptibility to disaster impacts.

Population Growth
This dimension measures demographic vitality and long-term socioeconomic development. The natural population growth rate is used to represent population dynamics that may influence community resilience and adaptive capacity.

Education
This dimension reflects human capital, risk awareness, and the ability to access and process disaster-related information. The proportion of females without formal education and the illiteracy rate are used as indicators of educational disadvantage. Higher educational attainment is generally associated with lower social vulnerability.

Healthcare Capacity
This dimension measures the availability of medical resources and access to health services. The number of health technicians per 1,000 people serves as an indicator of a community's ability to maintain public health and respond to disaster-related needs.

Infrastructure
This dimension reflects the availability and quality of essential public services and lifeline systems. Indicators include urban water supply penetration, urban gas penetration, and per capita electricity generation capacity. Stronger infrastructure generally enhances adaptive capacity and reduces vulnerability.

Transportation Accessibility
This dimension measures the accessibility of transportation resources that support mobility, evacuation, emergency response, and access to services. Public buses per 10,000 people are used as an indicator of transportation capacity, with higher accessibility contributing to lower social vulnerability.

Table S4. The spatial distribution changes of urbanization level in China.

Province	2006	2007	2008	2009	2010	2011	2012	2013	2014	2015	2016	2017	2018	2019	2020
Beijing	0.66	0.69	0.70	0.72	0.73	0.74	0.75	0.77	0.78	0.80	0.82	0.85	0.87	0.91	0.90
Tianjin	0.50	0.52	0.55	0.60	0.64	0.66	0.68	0.70	0.71	0.72	0.74	0.76	0.76	0.75	0.77
Hebei	0.35	0.36	0.40	0.43	0.47	0.48	0.49	0.50	0.51	0.53	0.55	0.57	0.58	0.60	0.61
Shanxi	0.29	0.30	0.33	0.38	0.42	0.44	0.47	0.49	0.51	0.55	0.56	0.57	0.59	0.60	0.62
Inner Mongolia	0.36	0.38	0.41	0.47	0.51	0.53	0.56	0.57	0.59	0.61	0.62	0.65	0.66	0.66	0.67
Liaoning	0.39	0.41	0.44	0.46	0.50	0.54	0.56	0.59	0.60	0.63	0.63	0.64	0.66	0.66	0.68
Jilin	0.31	0.34	0.40	0.44	0.46	0.49	0.49	0.52	0.54	0.55	0.57	0.57	0.60	0.61	0.63
Heilongjiang	0.27	0.34	0.34	0.37	0.38	0.39	0.43	0.49	0.52	0.58	0.61	0.62	0.63	0.64	0.66
Shanghai	0.64	0.65	0.68	0.73	0.70	0.72	0.75	0.75	0.78	0.80	0.81	0.82	0.84	0.87	0.87
Jiangsu	0.46	0.48	0.49	0.51	0.55	0.58	0.59	0.61	0.64	0.66	0.68	0.69	0.71	0.73	0.73
Zhejiang	0.44	0.47	0.49	0.51	0.54	0.56	0.58	0.60	0.61	0.63	0.65	0.68	0.69	0.71	0.71
Anhui	0.31	0.39	0.42	0.43	0.45	0.46	0.48	0.50	0.51	0.53	0.55	0.56	0.57	0.60	0.62
Fujian	0.38	0.42	0.45	0.48	0.51	0.53	0.54	0.56	0.57	0.59	0.61	0.63	0.65	0.67	0.69
Jiangxi	0.29	0.31	0.34	0.41	0.43	0.45	0.46	0.47	0.48	0.51	0.53	0.55	0.57	0.59	0.61
Shandong	0.39	0.43	0.45	0.47	0.50	0.52	0.54	0.56	0.57	0.59	0.61	0.62	0.64	0.64	0.65
Henan	0.27	0.32	0.36	0.39	0.41	0.42	0.44	0.45	0.48	0.51	0.53	0.54	0.56	0.58	0.59
Hubei	0.34	0.39	0.42	0.44	0.46	0.49	0.50	0.53	0.55	0.56	0.58	0.60	0.61	0.65	0.65
Hunan	0.33	0.35	0.37	0.39	0.43	0.46	0.48	0.50	0.52	0.54	0.56	0.58	0.59	0.61	0.63
Guangdong	0.38	0.43	0.48	0.51	0.55	0.56	0.58	0.60	0.61	0.64	0.65	0.67	0.68	0.71	0.71
Guangxi	0.30	0.32	0.37	0.40	0.43	0.39	0.46	0.47	0.48	0.50	0.52	0.54	0.56	0.59	0.60
Hainan	0.46	0.45	0.46	0.46	0.43	0.51	0.52	0.53	0.54	0.56	0.58	0.61	0.63	0.65	0.67
Chongqing	0.34	0.43	0.46	0.48	0.49	0.51	0.52	0.55	0.57	0.59	0.61	0.62	0.64	0.65	0.67
Sichuan	0.29	0.32	0.36	0.38	0.40	0.42	0.44	0.46	0.48	0.51	0.53	0.56	0.58	0.60	0.61
Guizhou	0.23	0.26	0.28	0.34	0.42	0.44	0.46	0.47	0.48	0.51	0.52	0.53	0.55	0.57	0.59
Yunnan	0.36	0.35	0.38	0.42	0.45	0.47	0.48	0.48	0.49	0.50	0.51	0.53	0.54	0.57	0.59
Tibet	0.20	0.25	0.24	0.25	0.25	0.23	0.27	0.18	0.30	0.23	0.47	0.50	0.48	0.52	0.52
Shaanxi	0.30	0.30	0.33	0.38	0.41	0.45	0.48	0.50	0.51	0.53	0.55	0.56	0.58	0.61	0.63
Gansu	0.29	0.31	0.33	0.35	0.36	0.39	0.42	0.45	0.47	0.51	0.53	0.55	0.56	0.57	0.58
Qinghai	0.27	0.30	0.31	0.33	0.33	0.40	0.40	0.42	0.43	0.45	0.51	0.52	0.56	0.60	0.61
Ningxia	0.33	0.34	0.39	0.36	0.46	0.46	0.52	0.54	0.54	0.56	0.58	0.60	0.61	0.63	0.63
Xinjiang	0.38	0.38	0.41	0.43	0.43	0.45	0.49	0.51	0.52	0.54	0.55	0.56	0.59	0.62	0.63

Table S5. Flood resilience evaluation results of 31 provinces in China from 2006 to 2020.

Province	2006	2007	2008	2009	2010	2011	2012	2013	2014	2015	2016	2017	2018	2019	2020
Beijing	0.40	0.41	0.42	0.43	0.46	0.46	0.47	0.48	0.49	0.52	0.55	0.55	0.57	0.40	0.56
Tianjin	0.38	0.39	0.39	0.40	0.41	0.42	0.42	0.44	0.44	0.45	0.47	0.46	0.45	0.44	0.45
Hebei	0.38	0.39	0.40	0.42	0.43	0.45	0.47	0.48	0.49	0.51	0.53	0.54	0.57	0.58	0.61
Shanxi	0.30	0.31	0.32	0.33	0.34	0.37	0.39	0.42	0.43	0.44	0.45	0.45	0.47	0.48	0.49
Inner Mongolia	0.30	0.32	0.34	0.37	0.39	0.42	0.44	0.46	0.46	0.48	0.48	0.48	0.49	0.50	0.51
Liaoning	0.39	0.39	0.41	0.42	0.44	0.46	0.48	0.50	0.49	0.48	0.49	0.49	0.50	0.52	0.52
Jilin	0.36	0.37	0.38	0.39	0.41	0.42	0.42	0.44	0.43	0.44	0.46	0.46	0.46	0.47	0.47
Heilongjiang	0.38	0.38	0.39	0.40	0.41	0.43	0.45	0.45	0.44	0.46	0.48	0.48	0.48	0.50	0.51
Shanghai	0.42	0.43	0.44	0.45	0.46	0.48	0.48	0.49	0.50	0.54	0.55	0.57	0.59	0.58	0.58
Jiangsu	0.42	0.43	0.45	0.48	0.50	0.55	0.56	0.57	0.59	0.65	0.65	0.66	0.70	0.70	0.73
Zhejiang	0.40	0.40	0.41	0.43	0.45	0.47	0.47	0.49	0.50	0.56	0.57	0.57	0.61	0.64	0.64
Anhui	0.35	0.35	0.38	0.39	0.40	0.43	0.45	0.48	0.47	0.52	0.52	0.53	0.55	0.56	0.56
Fujian	0.37	0.36	0.37	0.38	0.40	0.40	0.42	0.44	0.45	0.49	0.50	0.50	0.50	0.50	0.51
Jiangxi	0.33	0.34	0.36	0.37	0.37	0.41	0.43	0.44	0.45	0.48	0.49	0.50	0.51	0.52	0.53
Shandong	0.37	0.40	0.41	0.42	0.45	0.49	0.52	0.54	0.54	0.58	0.59	0.61	0.64	0.65	0.66
Henan	0.37	0.38	0.39	0.41	0.43	0.46	0.48	0.50	0.51	0.53	0.57	0.59	0.61	0.63	0.64
Hubei	0.36	0.36	0.39	0.40	0.41	0.44	0.46	0.49	0.49	0.54	0.54	0.58	0.56	0.58	0.59
Hunan	0.28	0.28	0.31	0.33	0.35	0.39	0.41	0.43	0.45	0.49	0.50	0.55	0.55	0.58	0.58
Guangdong	0.41	0.41	0.45	0.46	0.49	0.52	0.57	0.61	0.64	0.73	0.76	0.78	0.79	0.82	0.83
Guangxi	0.27	0.28	0.31	0.33	0.35	0.38	0.42	0.42	0.44	0.46	0.49	0.48	0.48	0.50	0.51
Hainan	0.34	0.32	0.33	0.33	0.34	0.34	0.35	0.37	0.36	0.37	0.37	0.40	0.38	0.44	0.41
Chongqing	0.30	0.32	0.32	0.35	0.36	0.41	0.43	0.44	0.45	0.49	0.48	0.50	0.48	0.48	0.49
Sichuan	0.36	0.37	0.41	0.42	0.44	0.46	0.47	0.53	0.55	0.56	0.58	0.60	0.61	0.62	0.65
Guizhou	0.22	0.21	0.24	0.25	0.27	0.30	0.33	0.35	0.40	0.42	0.43	0.44	0.44	0.46	0.47
Yunnan	0.30	0.32	0.34	0.36	0.37	0.40	0.41	0.44	0.46	0.47	0.50	0.52	0.51	0.54	0.54
Tibet	0.21	0.23	0.24	0.26	0.27	0.30	0.32	0.36	0.34	0.35	0.35	0.37	0.35	0.53	0.41
Shaanxi	0.31	0.34	0.35	0.37	0.39	0.41	0.43	0.44	0.45	0.46	0.47	0.50	0.50	0.50	0.51
Gansu	0.27	0.28	0.30	0.31	0.33	0.34	0.39	0.42	0.41	0.43	0.44	0.44	0.46	0.45	0.47
Qinghai	0.23	0.24	0.25	0.26	0.27	0.30	0.33	0.36	0.37	0.39	0.39	0.39	0.39	0.40	0.40
Ningxia	0.28	0.28	0.29	0.30	0.31	0.32	0.34	0.36	0.36	0.36	0.37	0.38	0.40	0.39	0.41
Xinjiang	0.31	0.32	0.33	0.34	0.36	0.37	0.39	0.41	0.42	0.43	0.44	0.46	0.48	0.50	0.50

Table S6. Social vulnerability evaluation results of 31 provinces in China from 2006 to 2020.

Province	2006	2007	2008	2009	2010	2011	2012	2013	2014	2015	2016	2017	2018	2019	2020
Beijing	0.27	0.28	0.26	0.24	0.25	0.22	0.20	0.18	0.15	0.13	0.11	0.08	0.10	0.08	0.07
Tianjin	0.41	0.40	0.38	0.37	0.35	0.32	0.29	0.25	0.24	0.21	0.18	0.16	0.17	0.16	0.13
Hebei	0.78	0.75	0.73	0.72	0.70	0.68	0.66	0.64	0.63	0.60	0.57	0.54	0.52	0.51	0.49
Shanxi	0.76	0.74	0.72	0.70	0.68	0.65	0.62	0.60	0.59	0.55	0.54	0.51	0.50	0.48	0.46
Inner Mongolia	0.71	0.68	0.67	0.64	0.62	0.59	0.57	0.45	0.50	0.48	0.46	0.43	0.42	0.41	0.38
Liaoning	0.59	0.58	0.56	0.55	0.53	0.49	0.47	0.45	0.42	0.40	0.40	0.39	0.37	0.35	0.33
Jilin	0.66	0.65	0.64	0.63	0.63	0.61	0.60	0.57	0.56	0.54	0.54	0.52	0.50	0.49	0.47
Heilongjiang	0.66	0.65	0.62	0.62	0.61	0.59	0.58	0.55	0.53	0.51	0.50	0.48	0.46	0.44	0.42
Shanghai	0.25	0.25	0.23	0.22	0.22	0.18	0.18	0.16	0.14	0.14	0.12	0.10	0.10	0.08	0.07
Jiangsu	0.65	0.60	0.58	0.57	0.52	0.48	0.46	0.42	0.38	0.34	0.30	0.26	0.26	0.23	0.20
Zhejiang	0.60	0.56	0.54	0.53	0.49	0.46	0.44	0.42	0.39	0.36	0.33	0.30	0.29	0.26	0.24
Anhui	0.80	0.78	0.75	0.74	0.72	0.69	0.67	0.65	0.63	0.61	0.58	0.55	0.54	0.52	0.50
Fujian	0.69	0.66	0.63	0.61	0.56	0.54	0.51	0.50	0.47	0.45	0.42	0.39	0.38	0.36	0.34
Jiangxi	0.78	0.76	0.74	0.72	0.71	0.68	0.66	0.64	0.62	0.59	0.56	0.53	0.52	0.50	0.48
Shandong	0.69	0.67	0.66	0.65	0.63	0.61	0.58	0.56	0.54	0.50	0.46	0.42	0.42	0.40	0.38
Henan	0.85	0.83	0.81	0.79	0.78	0.75	0.72	0.70	0.68	0.65	0.62	0.59	0.57	0.54	0.53
Hubei	0.72	0.71	0.69	0.69	0.66	0.62	0.60	0.58	0.56	0.54	0.51	0.49	0.48	0.46	0.44
Hunan	0.78	0.75	0.73	0.72	0.72	0.69	0.67	0.65	0.63	0.59	0.57	0.46	0.52	0.40	0.47
Guangdong	0.57	0.55	0.53	0.52	0.49	0.47	0.45	0.44	0.41	0.39	0.37	0.35	0.34	0.33	0.30
Guangxi	0.83	0.80	0.78	0.76	0.75	0.73	0.70	0.68	0.66	0.64	0.62	0.60	0.58	0.57	0.55
Hainan	0.73	0.72	0.70	0.68	0.67	0.64	0.62	0.60	0.58	0.56	0.53	0.50	0.50	0.48	0.47
Chongqing	0.72	0.68	0.65	0.64	0.63	0.59	0.56	0.54	0.52	0.50	0.47	0.44	0.43	0.41	0.39
Sichuan	0.83	0.80	0.78	0.76	0.75	0.71	0.69	0.66	0.64	0.62	0.59	0.56	0.55	0.52	0.50
Guizhou	0.91	0.90	0.88	0.87	0.84	0.81	0.80	0.77	0.73	0.69	0.66	0.63	0.61	0.58	0.56
Yunnan	0.87	0.84	0.83	0.82	0.81	0.78	0.76	0.74	0.72	0.70	0.67	0.65	0.63	0.62	0.59
Tibet	0.89	0.87	0.91	0.90	0.88	0.91	0.96	0.93	0.89	0.83	0.84	0.80	0.81	0.79	0.77
Shaanxi	0.78	0.75	0.73	0.71	0.69	0.65	0.62	0.60	0.58	0.56	0.54	0.43	0.50	0.47	0.45
Gansu	0.88	0.86	0.86	0.84	0.81	0.78	0.76	0.74	0.72	0.69	0.67	0.64	0.62	0.59	0.57
Qinghai	0.76	0.74	0.72	0.71	0.68	0.66	0.64	0.63	0.61	0.59	0.56	0.53	0.51	0.49	0.47
Ningxia	0.76	0.74	0.72	0.70	0.67	0.65	0.64	0.60	0.57	0.54	0.52	0.48	0.47	0.45	0.43
Xinjiang	0.78	0.75	0.75	0.75	0.71	0.69	0.68	0.67	0.64	0.61	0.59	0.58	0.55	0.53	0.51

Table S7. Ternary CCD for the three systems in 31 provinces of China from 2006 to 2020.

Province	2006	2007	2008	2009	2010	2011	2012	2013	2014	2015	2016	2017	2018	2019	2020
Beijing	0.46	0.47	0.48	0.49	0.52	0.52	0.53	0.53	0.54	0.57	0.59	0.60	0.62	0.49	0.61
Tianjin	0.40	0.41	0.43	0.45	0.47	0.48	0.48	0.49	0.50	0.50	0.52	0.51	0.51	0.49	0.50
Hebei	0.26	0.29	0.32	0.33	0.34	0.36	0.38	0.40	0.40	0.43	0.46	0.48	0.51	0.51	0.54
Shanxi	0.26	0.28	0.29	0.30	0.32	0.35	0.37	0.40	0.41	0.43	0.44	0.45	0.47	0.48	0.49
Inner Mongolia	0.29	0.31	0.33	0.35	0.37	0.40	0.42	0.48	0.46	0.47	0.49	0.49	0.50	0.51	0.53
Liaoning	0.38	0.40	0.42	0.43	0.44	0.47	0.49	0.51	0.51	0.50	0.51	0.52	0.53	0.55	0.56
Jilin	0.32	0.34	0.37	0.37	0.38	0.39	0.40	0.42	0.42	0.43	0.45	0.45	0.45	0.46	0.47
Heilongjiang	0.29	0.34	0.35	0.37	0.38	0.40	0.42	0.44	0.44	0.45	0.47	0.48	0.48	0.50	0.52
Shanghai	0.46	0.47	0.48	0.50	0.50	0.51	0.53	0.53	0.54	0.58	0.59	0.61	0.63	0.63	0.63
Jiangsu	0.37	0.40	0.42	0.45	0.48	0.52	0.54	0.57	0.60	0.65	0.65	0.66	0.69	0.69	0.72
Zhejiang	0.39	0.41	0.42	0.43	0.46	0.49	0.50	0.52	0.53	0.58	0.59	0.59	0.63	0.65	0.65
Anhui	0.23	0.26	0.29	0.30	0.32	0.35	0.37	0.39	0.40	0.43	0.45	0.47	0.48	0.50	0.51
Fujian	0.33	0.34	0.36	0.37	0.40	0.41	0.44	0.45	0.47	0.50	0.52	0.53	0.53	0.53	0.54
Jiangxi	0.24	0.26	0.29	0.31	0.31	0.35	0.37	0.39	0.40	0.43	0.45	0.47	0.48	0.50	0.51
Shandong	0.33	0.35	0.36	0.37	0.39	0.42	0.45	0.46	0.48	0.52	0.56	0.59	0.59	0.61	0.63
Henan	0.19	0.22	0.24	0.26	0.28	0.30	0.32	0.34	0.37	0.39	0.42	0.45	0.46	0.48	0.50
Hubei	0.30	0.31	0.33	0.34	0.36	0.39	0.42	0.44	0.45	0.48	0.50	0.53	0.53	0.54	0.56
Hunan	0.24	0.26	0.28	0.29	0.30	0.33	0.35	0.37	0.39	0.42	0.45	0.54	0.50	0.58	0.54
Guangdong	0.39	0.42	0.46	0.46	0.49	0.52	0.55	0.57	0.60	0.60	0.61	0.63	0.64	0.65	0.67
Guangxi	0.21	0.22	0.25	0.27	0.28	0.31	0.33	0.35	0.37	0.38	0.41	0.42	0.43	0.44	0.45
Hainan	0.28	0.28	0.30	0.31	0.32	0.33	0.34	0.36	0.36	0.38	0.39	0.41	0.40	0.44	0.42
Chongqing	0.28	0.30	0.32	0.34	0.35	0.40	0.42	0.43	0.45	0.48	0.48	0.50	0.49	0.50	0.51
Sichuan	0.21	0.24	0.26	0.28	0.30	0.33	0.35	0.37	0.39	0.41	0.44	0.47	0.48	0.51	0.53
Guizhou	0.14	0.14	0.16	0.17	0.19	0.21	0.24	0.26	0.30	0.34	0.36	0.38	0.39	0.41	0.43
Yunnan	0.18	0.21	0.22	0.23	0.24	0.27	0.29	0.31	0.33	0.35	0.38	0.40	0.41	0.43	0.44
Tibet	0.14	0.17	0.14	0.15	0.16	0.13	0.10	0.10	0.17	0.18	0.21	0.24	0.23	0.29	0.28
Shaanxi	0.25	0.27	0.29	0.32	0.34	0.36	0.39	0.41	0.43	0.44	0.45	0.52	0.49	0.50	0.51
Gansu	0.17	0.18	0.19	0.21	0.24	0.26	0.29	0.31	0.32	0.34	0.35	0.37	0.39	0.40	0.42
Qinghai	0.23	0.25	0.26	0.27	0.29	0.31	0.34	0.36	0.37	0.39	0.40	0.41	0.41	0.42	0.43
Ningxia	0.25	0.26	0.27	0.29	0.30	0.32	0.33	0.36	0.37	0.38	0.39	0.41	0.42	0.43	0.44
Xinjiang	0.25	0.27	0.27	0.27	0.30	0.32	0.33	0.35	0.37	0.39	0.40	0.42	0.44	0.46	0.47

Table S8. Standards for determining CCD between urbanization, flood resilience and SOVI systems.

Classes	Ranges of CCD	Coordination Level
Balanced development	0.8< D≤1	Superiorly coordinated
(Acceptable interval)	0.6< D≤ 0.8	Coordinated development
Transitional development	0.5< D≤ 0.6	Barely coordinated
(Transitional interval	0.4< D≤ 0.5	Slightly uncoordinated
Unbalanced development	0.2< D≤ 0.4	Moderately uncoordinated
(Unacceptable interval)	0< D ≤ 0.2	Seriously uncoordinated

Table S9. Changes in the three systems in each province.

Province	2006	2007	2008	2009	2010	2011	2012	2013	2014	2015	2016	2017	2018	2019	2020
**Beijing**	H-H	H-H	H-H	H-H	H-H	H-H	H-H	H-H	H-H	H-H	H-H	H-H	H-H	L-L	H-L
	U	U	U	U	U	U	U	U	U	U	U	U	U	U	U
	0.46	0.47	0.48	0.49	0.52	0.52	0.53	0.53	0.54	0.57	0.59	0.60	0.62	0.49	0.61
	3	3	3	3	4	4	4	4	4	4	4	4	5	3	5
**Tianjin**	H-H	H-H	H-H	H-H	H-H	H-H	H-H	H-H	H-H	H-H	H-H	H-H	H-H	L-L	L-H
	U	U	U	U	U	U	U	U	U	U	U	U	U	U	U
	0.40	0.41	0.43	0.45	0.47	0.48	0.48	0.49	0.50	0.50	0.52	0.51	0.51	0.49	0.50
	3	3	3	3	3	3	3	3	3	4	4	4	4	3	4
**Hebei**	L-H	L-H	L-H	L-H	L-H	L-H	L-H	L-H	L-H	L-H	L-H	L-H	H-H	H-H	H-H
	S	S	S	S	S	S	S	S	S	S	S	U	R	R	R
	0.26	0.29	0.32	0.33	0.34	0.36	0.38	0.40	0.40	0.43	0.46	0.48	0.51	0.51	0.54
	2	2	2	2	2	2	2	2	3	3	3	3	4	4	4
**Shanxi**	L-L	L-L	L-L	L-L	L-L	L-L	L-L	L-L	L-L	L-H	L-L	L-L	L-L	L-L	L-L
	S	S	S	S	S	S	S	S	S	S	S	U	U	U	U
	0.26	0.28	0.29	0.30	0.32	0.35	0.37	0.40	0.41	0.43	0.44	0.45	0.47	0.48	0.49
	2	2	2	2	2	2	2	2	3	3	3	3	3	3	3
**Inner Mongolia**	H-L	H-L	H-L	H-L	H-L	H-L	H-L	H-L	H-L	H-L	H-L	H-L	H-L	H-L	H-L
	S	S	S	S	S	S	S	U	U	U	U	U	U	U	U
	0.29	0.31	0.33	0.35	0.37	0.40	0.42	0.48	0.46	0.47	0.49	0.49	0.50	0.51	0.53
	2	2	2	2	2	2	3	3	3	3	3	3	4	4	4
**Liaoning**	H-H	H-H	H-H	H-H	H-H	H-H	H-H	H-H	H-H	H-L	H-L	H-L	H-L	H-L	H-L
	S	S	S	S	S	U	U	U	U	U	U	U	U	U	U
	0.38	0.40	0.42	0.43	0.44	0.47	0.49	0.51	0.51	0.50	0.51	0.52	0.53	0.55	0.56
	2	2	3	3	3	3	3	4	4	4	4	4	4	4	4
**Jilin**	H-H	H-H	H-H	H-H	H-H	H-H	H-H	H-H	L-H	L-H	L-H	L-H	L-H	L-H	L-H
	S	S	S	S	S	S	S	S	S	S	U	U	U	U	U
	0.32	0.34	0.37	0.37	0.38	0.39	0.40	0.42	0.42	0.43	0.45	0.45	0.45	0.46	0.47
	2	2	2	2	2	2	3	3	3	3	3	3	3	3	3
**Heilongjiang**	H-H	H-H	H-H	H-H	H-H	H-H	H-H	H-H	H-H	H-H	H-H	L-L	L-L	L-L	L-L
	S	S	S	S	S	S	S	S	S	U	U	U	U	U	U
	0.29	0.34	0.35	0.37	0.38	0.40	0.42	0.44	0.44	0.45	0.47	0.48	0.48	0.50	0.52
	2	2	2	2	2	2	3	3	3	3	3	3	3	4	4
**Shanghai**	H-H	H-H	H-H	H-H	H-H	H-H	H-H	H-H	H-H	H-H	H-H	H-H	H-H	H-H	H-H
	U	U	U	U	U	U	U	U	U	U	U	U	U	U	U
	0.46	0.47	0.48	0.50	0.50	0.51	0.53	0.53	0.54	0.58	0.59	0.61	0.63	0.63	0.63

	3	3	3	4	4	4	4	4	4	4	4	5	5	5	5
**Jiangsu**	H-H	H-H	H-H	H-H	H-H	H-H	H-H	H-H	H-H	H-H	H-H	H-H	H-H	H-H	H-H
	S	S	S	S	S	R	R	U	U	R	R	R	R	R	R
	0.37	0.40	0.42	0.45	0.48	0.52	0.54	0.57	0.60	0.65	0.65	0.66	0.69	0.69	0.72
	2	3	3	3	3	4	4	4	4	5	5	5	5	5	5
**Zhejiang**	H-H	H-H	H-H	H-H	H-H	H-H	H-H	H-H	H-H	H-H	H-H	H-H	H-H	H-H	H-H
	S	S	S	S	U	U	U	U	U	U	U	U	U	U	U
	0.39	0.41	0.42	0.43	0.46	0.49	0.50	0.52	0.53	0.58	0.59	0.59	0.63	0.65	0.65
	2	3	3	3	3	3	3	4	4	4	4	4	5	5	5
**Anhui**	L-H	L-H	L-H	L-H	L-H	L-H	L-H	L-H	L-H	L-H	L-H	L-H	L-H	L-H	L-H
	S	S	S	S	S	S	S	S	S	S	S	S	R	U	U
	0.23	0.26	0.29	0.30	0.32	0.35	0.37	0.39	0.40	0.43	0.45	0.47	0.48	0.50	0.51
	2	2	2	2	2	2	2	2	3	3	3	3	3	3	4
**Fujian**	H-H	H-H	H-H	H-H	H-H	H-H	H-H	H-H	H-H	H-H	H-H	H-H	H-H	H-H	H-H
	S	S	S	S	S	S	S	U	U	U	U	U	U	U	U
	0.33	0.34	0.36	0.37	0.40	0.41	0.44	0.45	0.47	0.50	0.52	0.53	0.53	0.53	0.54
	2	2	2	2	3	3	3	3	3	4	4	4	4	4	4
**Jiangxi**	L-H	L-H	L-H	L-H	L-H	L-H	L-H	L-H	L-H	L-H	L-H	L-H	L-H	L-H	L-H
	S	S	S	S	S	S	S	S	S	S	S	S	U	U	U
	0.24	0.26	0.29	0.31	0.31	0.35	0.37	0.39	0.40	0.43	0.45	0.47	0.48	0.50	0.51
	2	2	2	2	2	2	2	2	2	3	3	3	3	3	4
**Shandong**	H-L	H-L	H-L	H-H	H-H	H-H	H-H	H-H	H-H	H-H	H-H	H-H	H-H	H-H	H-H
	S	S	S	S	S	S	S	S	U	R	R	R	R	R	R
	0.33	0.35	0.36	0.37	0.39	0.42	0.45	0.46	0.48	0.52	0.56	0.59	0.59	0.61	0.63
	2	2	2	2	2	3	3	3	3	4	4	4	4	5	5
**Henan**	L-L	L-L	L-L	L-L	L-L	L-L	L-H	L-H	L-H	L-H	L-H	L-H	L-H	L-H	L-H
	S	S	S	S	S	S	S	S	S	S	S	R	R	R	R
	0.19	0.22	0.24	0.26	0.28	0.30	0.32	0.34	0.37	0.39	0.42	0.45	0.46	0.48	0.50
	1	2	2	2	2	2	2	2	2	2	3	3	3	3	4
**Hubei**	H-L	H-L	H-L	H-L	H-L	H-L	H-L	H-L	H-L	H-L	H-L	H-H	H-L	H-H	H-L
	S	S	S	S	S	S	S	S	S	R	U	R	U	U	U
	0.30	0.31	0.33	0.34	0.36	0.39	0.42	0.44	0.45	0.48	0.50	0.53	0.53	0.54	0.56
	2	2	2	2	2	2	3	3	3	3	4	4	4	4	4
**Hunan**	L-L	L-L	L-L	L-L	L-L	L-L	L-L	L-L	L-L	L-L	L-L	H-L	H-L	H-L	H-L
	S	S	S	S	S	S	S	S	S	S	S	R	U	R	U
	0.24	0.26	0.28	0.29	0.30	0.33	0.35	0.37	0.39	0.42	0.45	0.54	0.50	0.58	0.54
	2	2	2	2	2	2	2	2	2	3	3	4	3	4	4

**Guangdong**	H-L	H-L	H-L	H-L	H-L	H-L	H-L	H-L	H-L	H-L	H-L	H-L	H-L	H-L	H-L
	S	S	S	S	U	U	R	R	R	R	R	R	R	R	R
	0.39	0.42	0.46	0.46	0.49	0.52	0.55	0.57	0.60	0.60	0.61	0.63	0.64	0.65	0.67
	2	3	3	3	3	4	4	4	4	4	5	5	5	5	5
**Guangxi**	L-L	L-L	L-L	L-L	L-L	L-L	L-L	L-L	L-L	L-L	L-L	L-H	L-L	L-H	L-H
	S	S	S	S	S	S	S	S	S	S	S	S	S	S	U
	0.21	0.22	0.25	0.27	0.28	0.31	0.33	0.35	0.37	0.38	0.41	0.42	0.43	0.44	0.45
	2	2	2	2	2	2	2	2	2	2	3	3	3	3	3
**Chongqing**	H-L	H-L	H-L	H-L	L-L	H-L	H-L	H-L	H-L	H-L	H-L	H-H	L-L	L-H	L-L
	S	S	S	S	S	S	S	S	U	U	U	U	U	U	U
	0.28	0.30	0.32	0.34	0.35	0.40	0.42	0.43	0.45	0.48	0.48	0.50	0.49	0.50	0.51
	2	2	2	2	2	2	3	3	3	3	3	4	3	4	4
**Sichuan**	L-L	L-L	L-L	L-L	L-L	L-L	L-L	L-L	L-L	L-L	L-L	L-L	L-L	H-L	H-L
	S	S	S	S	S	S	S	S	S	S	R	R	R	R	R
	0.21	0.24	0.26	0.28	0.30	0.33	0.35	0.37	0.39	0.41	0.44	0.47	0.48	0.51	0.53
	2	2	2	2	2	2	2	2	2	3	3	3	3	4	4
**Guizhou**	L-L	L-L	L-L	L-L	L-L	L-L	L-L	L-L	L-L	L-L	L-L	L-L	L-L	L-L	L-L
	S	S	S	S	S	S	S	S	S	S	S	S	S	S	U
	0.14	0.14	0.16	0.17	0.19	0.21	0.24	0.26	0.30	0.34	0.36	0.38	0.39	0.41	0.43
	1	1	1	1	1	2	2	2	2	2	2	2	2	3	3
**Yunnan**	L-L	L-L	L-L	L-L	L-L	L-L	L-L	L-L	L-L	L-L	L-L	L-L	L-L	L-L	L-L
	S	S	S	S	S	S	S	S	S	S	S	S	S	S	S
	0.18	0.21	0.22	0.23	0.24	0.27	0.29	0.31	0.33	0.35	0.38	0.40	0.41	0.43	0.44
	1	2	2	2	2	2	2	2	2	2	2	2	3	3	3
**Tibet**	L-L	L-L	L-L	L-L	L-L	L-L	L-L	L-L	L-L	L-L	L-L	L-L	L-L	L-L	L-L
	S	S	S	S	S	S	S	S	S	S	S	S	S	S	S
	0.14	0.17	0.14	0.15	0.16	0.13	0.10	0.10	0.17	0.18	0.21	0.24	0.23	0.29	0.28
	1	1	1	1	1	1	1	1	1	1	2	2	2	2	2
**Shaanxi**	L-L	L-L	L-L	L-L	L-L	L-L	H-L	L-L	L-L	L-L	L-L	H-L	L-L	L-L	L-L
	S	S	S	S	S	S	S	S	S	S	S	U	U	U	U
	0.25	0.27	0.29	0.32	0.34	0.36	0.39	0.41	0.43	0.44	0.45	0.52	0.49	0.50	0.51
	2	2	2	2	2	2	2	3	3	3	3	4	3	3	4
**Gansu**	L-L	L-L	L-L	L-L	L-L	L-L	L-L	L-L	L-L	L-L	L-L	L-L	L-L	L-L	L-L
	S	S	S	S	S	S	S	S	S	S	S	S	S	S	S
	0.17	0.18	0.19	0.21	0.24	0.26	0.29	0.31	0.32	0.34	0.35	0.37	0.39	0.40	0.42
	1	1	1	2	2	2	2	2	2	2	2	2	2	3	3
**Qinghai**	L-L	L-L	L-L	L-L	L-L	L-L	L-L	L-L	L-L	L-L	L-L	L-L	L-L	L-L	L-L

	S	S	S	S	S	S	S	S	S	S	U	U	U	U	U
	0.23	0.25	0.26	0.27	0.29	0.31	0.34	0.36	0.37	0.39	0.40	0.41	0.41	0.42	0.43
	2	2	2	2	2	2	2	2	2	2	2	3	3	3	3
**Ningxia**	L-L	L-L	L-L	L-L	L-L	L-L	L-L	L-L	L-L	L-L	L-L	L-L	L-L	L-L	L-L
	S	S	S	S	S	S	S	S	S	S	S	S	U	U	U
	0.25	0.26	0.27	0.29	0.30	0.32	0.33	0.36	0.37	0.38	0.39	0.41	0.42	0.43	0.44
	2	2	2	2	2	2	2	2	2	2	2	3	3	3	3
**Xinjiang**	L-L	L-L	L-L	L-L	L-L	L-L	L-L	L-L	L-L	L-L	L-L	L-L	L-L	L-L	L-L
	S	S	S	S	S	S	S	S	S	S	S	S	U	U	U
	0.25	0.27	0.27	0.27	0.30	0.32	0.33	0.35	0.37	0.39	0.40	0.42	0.44	0.46	0.47
	2	2	2	2	2	2	2	2	2	2	3	3	3	3	3

Dataset S1. Index dataset for urbanization, flood resilience and social vulnerability system.
The data and code are available on Zenodo at DOI: 10.5281/zenodo.18253894.
https://zenodo.org/records/18253895

SI References

1. B. Neumann, A. T. Vafeidis, J. Zimmermann, R. J. Nicholls, Future coastal population growth and exposure to sea-level rise and coastal flooding—a global assessment. *PLOS ONE* **10**, e0131375 (2015).
2. Y. Liu, X. Huang, H. Yang, An integrated approach to investigate the coupling coordination between urbanization and flood disasters in China. *Journal of Cleaner Production* **375**, 134191 (2022).
3. H. Miao, N. Wang, Y. Wang, P. Lin, An urban resilience measurement system based on decomposing post-disaster recovery process (in Chinese). *Journal of Natural Disasters* **30**, 10–27 (2021).
4. Y. Liu, L. Yang, W. Jiang, Coupling coordination and spatiotemporal dynamic evolution between social economy and water environmental quality—a case study from Nansi Lake catchment, China. *Ecological Indicators* **119**, 106870 (2020).
5. Z. Zhang, Y. Li, Coupling coordination and spatiotemporal dynamic evolution between urbanization and geological hazards—a case study from China. *Science of the Total Environment* **728**, 138825 (2020).
